# Tuning the auxiliary donor in D–D–π–A photosensitizers to enhance DSSC photovoltaic performance: a DFT/TDDFT study

**DOI:** 10.1039/d5ra07959d

**Published:** 2025-12-17

**Authors:** Rinki Deka, Bikash Kalita, Dhruba Jyoti Kalita

**Affiliations:** a Department of Chemistry, Gauhati University Guwahati-781014 India dhrubajyoti.kalita@gauhati.ac.in

## Abstract

Harvesting solar energy from sunlight to generate electricity is considered one of the most important technologies to address future sustainability. Dye-sensitized solar cells (DSSCs) have attracted tremendous interest and attention over the past two decades due to their potential advantages for being implemented on large areas and using light-weight flexible substrates. The electronic, optical, and photovoltaic properties of dyes are pivotal for efficient solar energy conversion, and these properties can be finely tuned by structural modifications. In this study, we have designed a series of D–D–π–A architectured dyes, employing coumarin–thiophene–cyanoacrylic acid as the D–π–A core with the integration of eight efficient auxiliary donor units. Density functional theory (DFT) and time-dependent DFT (TD-DFT) computations have been employed to elucidate the electronic structure and optical characteristics of the dyes. Through DFT and TD-DFT simulations, we investigate the impact of various auxiliary donors on geometrical configurations, electronic structures, and optical properties. Our findings reveal that the incorporation of double donors not only enhances electron-donating capabilities but also impedes aggregation between dye molecules, thereby preventing recombination of injected electrons with the I^−^/I_3_^−^ in the electrolyte at TiO_2_ semiconducting surfaces. This study underscores the effectiveness of incorporating auxiliary donor groups into organic dyes as a promising strategy for the development of high-performance, metal-free organic dyes tailored for photovoltaic applications.

## Abstract 2

Through DFT and TD-DFT simulations, we investigate the impact of various auxiliary donors on the geometrical configurations, electronic structures, and optical properties of a series of D–D–π–A architectured dyes.

## Graphical abstract

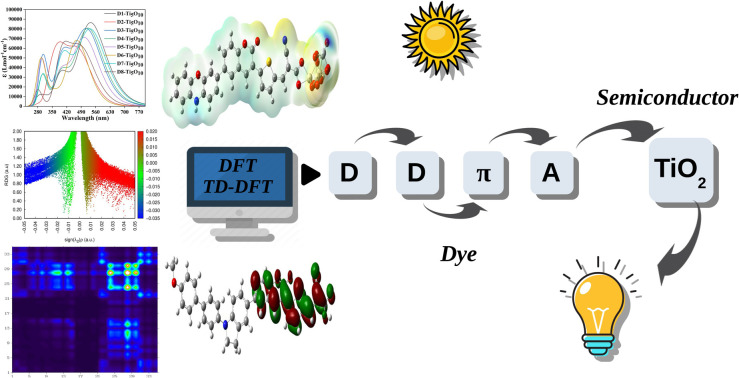

## 1 Introduction

Global energy consumption is increasing day by day to meet human requirements. In today's world, fossil fuels such as coal, natural gas, and oil are the primary sources of energy. However, they have several drawbacks, such as being non-renewable, more expensive and unsustainable, and causing environmental degradation.^1^ The world is now grappling with two major issues: the absence of sustainable, safe and environmentally acceptable energy sources, and environmental contamination.^2^ Due to the depletion of fossil fuels and the challenges associated with environmental contamination, there is a growing demand for alternative energy sources that are both environment friendly and sustainable.^3^ Nature has always used the sun as a reliable source of inexpensive and clean energy to support almost all life on earth.^4^ Renewable energy sources, like solar radiation, wind, hydro power, and geothermal heat, are described as energy harnessed from naturally replenished sources that continue to regenerate over time.^5^ Solar energy has clearly become the dominant trend in sustainable energy^6^ and can be turned directly into electrical energy through the use of photovoltaic devices.^7^ Solar energy is one of the most promising future energy resources considering its availability, cleanliness, safety and higher economic viability, allowing large-scale energy production. Photovoltaic cells are appealing because they do not pollute the environment, are inexhaustible, and are extremely effective. They also deliver clean and green energy, with lower expense, fuel-free operation, cheap maintenance, and so forth.^8^ Photovoltaic cells are categorized into three different generations depending on their working mechanism, production techniques and materials used.^9^ The first-generation solar cells include crystalline silicon-based solar cells. The power conversion efficiency (PCE) of these types of solar cells is in between 12–16% depending on the manufacturing procedures.^10^ The second-generation solar cells are based on thin-film technology. Thin-film solar cells have a lower manufacturing cost than silicon-based technology because of material savings, integrated cell insulation evolved at low temperatures, and highly automated series production.^11,12^ The third-generation solar cells have attracted much more attention in comparison to the first- and second-generation solar cells due to their high efficiency, low cost and easy manufacturing process.^13^ The third-generation solar cells are categorized as dye-sensitized solar cells (DSSCs),^14^ quantum-dot solar cells,^15^ bulk-heterojunction solar cells,^16^ perovskite solar cells,^17^ organic tandem solar cells,^18^ inorganic solar cells,^19^*etc.* Among these third-generation categories, DSSCs are extensively applicable and are almost on the edge of commercialization.^20^

DSSCs are semiconductor photovoltaic devices that directly transform solar radiation into electric current.^21^ A basic DSSC consists of a molecular dye, a mesoporous wide-band-gap semiconductor oxide, a redox couple electrolyte and a collector/counter electrode. The dye molecule (photosensitizer) absorbs sunlight and injects its excited electrons into the conduction band of the semiconductor and becomes oxidized. The redox electrolyte acts as an intermediate to transfer holes from the dye to the counter electrode for regeneration of the dye.^22^ Dyes with good light-harvesting ability and appropriate energy levels may lead to adequate photoelectric conversion efficiency.^23–25^ The most stable and efficient dyes for DSSCs are metal–organic dyes, such as those containing ruthenium and osmium, but they are costly, difficult to synthesize, and hazardous by nature. Owing to the drawbacks of metal organic dyes, novel donor–π–acceptor (D–π–A)-type metal-free organic dyes have been described to enhance the efficiency of DSSCs.^26^ Thus metal-free organic dyes have been synthesized and used as sensitizers in DSSCs because of their chemical adaptability, simple synthetic approaches to various molecular structures, ease of purification, low material cost, high molar extinction coefficients, and high levels of solar spectral absorption in the visible range.^27,28^ To increase the efficiency of DSSC devices, significant research and development is going into manufacturing DSSCs based on organic compounds with a variety of molecular structures, including D–π–A, D–A–A, D–π–D, A–π–A, and D–π–A–π–D. The D–π–A architecture can facilitate effective intramolecular charge transfer (ICT) from the donor group of the dye to its acceptor unit through the conjugated π-spacer, which is necessary for effective electron injection into the semiconductor.^29^ In the typical D–π–A architecture, the D units determine the optical characteristics of the dye. The molecular engineering of different donor units has led to the development of various sensitizers that enhance photovoltaic device performance and modulate the absorption characteristics of the dyes.^30^ Double donors may provide the sensitizers with an expanded absorption region, improved power conversion efficiency, higher molar extinction coefficients and increased light absorption capacities.^13^ In addition, double donors have influenced both molecular energy levels and light-harvesting ability. In 2008, Ning *et al.* introduced an innovative design for D–D–π–A structured dyes, employing a starburst triarylamine group as an electron donor. This configuration significantly boosted photovoltaic efficiency when compared to utilizing a single triphenylamine unit. Such an approach holds promise in enhancing the stability of solar cells.^31^ In addition to increasing light absorption capacity, the extra electron donor in the D–D–π–A structure also helps to prevent dye aggregation.^32,33^ Moreover, Dai *et al.* have designed an M45 sensitizer based on the D–D–π–A architecture that has an overall power conversion efficiency of 9.02%.^34^ Recently, Lin *et al.* designed double-donor-based dyes (ME101–ME106) with promising outcomes.^35^ These findings indicate that D–D–π–A-based sensitizers have significant benefits in DSSC fields.

**Fig. 1:**
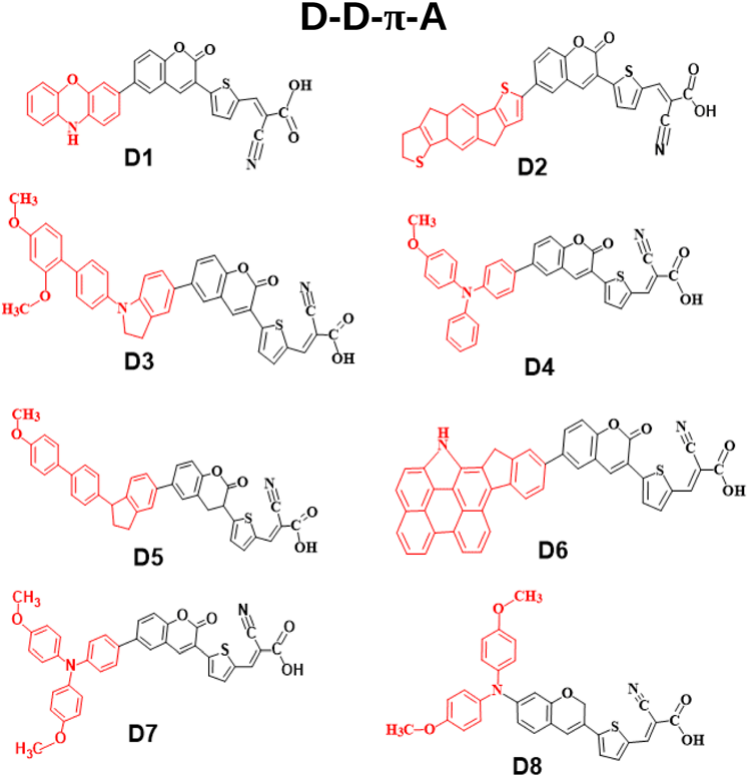
Structures of the designed dyes.

The work of Liang Han *et al.*, where they successfully synthesized a parent dye based on a triphenylamine-coumarin moiety, has inspired us to investigate the tuning of dye properties by modifying the auxiliary donor moiety.^13^ In this work, we have modified the end donor unit in a D–D–π–A-based architecture. In this context, we have utilized coumarin–thiophene–cyanoacrylic acid as the D–π–A component and integrated an additional end auxiliary donor unit for enhanced efficiency, *viz.*, phenoxazine (D1), indacenodithiophene (D2), dimethoxy-substituted indoline (D3), methoxy-substituted triphenyl amine (D4), methoxy-substituted indoline (D5), *N*-annulated indenoperylene (D6), dimethoxy-substituted triphenylamine (D7), and methoxy-substituted diphenyl amine (D8). We have used density functional theory (DFT) and time-dependent DFT (TD-DFT) to determine the electronic structure and optical absorption characteristics of the dyes. Based upon the obtained results, we have studied the role of different auxiliary donors in tuning the geometries, electronic structures, and optical properties. The key parameters, including dihedral angle, dipole moment, HOMO and LUMO energies with their differences (*Δ*_H–L_ values), ionization potential, electron affinity, ground-state oxidation potential, excited-state oxidation potential, light harvesting capacity, absorption properties, reorganization energies, charge transfer rate, *etc.*, have been extensively evaluated. Additionally, we have adopted systematic investigations to gauge the effect of the dye/TiO_2_ on the electronic and optical characteristics. To assess the performance of the adsorption systems in DSSCs, we have evaluated the geometry, electron injection, absorption spectra, and excited state of the dye/TiO_2_ cluster system. To the best of our knowledge, design and theoretical study of these molecules based on the D–D–π–A architecture have been reported very rarely. The obtained results offer significant guidance for developing novel dyes based on double donors that will result in highly efficient DSSCs. The structures of all the designed dyes are presented in Fig. 1.

## 2 Computational details and validation of methodology

The Gaussian 09 software package has been used for all of the computations.^36^ Density functional theory (DFT) has been employed to optimize the ground-state geometry, while time-dependent DFT (TD-DFT) has been used to calculate the excited-state properties. To validate the functionals used in our calculations, we have carried out an extensive study to improve the accuracy of the results. We have performed a test calculation with a coumarin-based dye reported in the literature because of its structural resemblance to our designed dyes.^13^ The DFT approach has been used to calculate the ground-state geometries using six distinct functionals: B3LYP, B3LYP-D3, CAM-B3LYP, B3PW91, wB97XD, and HSEH1PBE, along with the 6-31G(d) basis set. However, for the excited-state calculations, time-dependent density functional theory (TD-DFT) has been performed using the above-mentioned functionals and basis set. The optimized structure of the test compound has been presented in Fig. S1 (SI) and the calculated results have been reported in Table S2 (SI). We have compared the calculated *Δ*_H–L_ value and *λ*_max_ with the reported results. Table S2 (SI) shows that the values obtained using the B3LYP-D3/6-31G(d) functional/basis set correspond well with the experimental results for ground-state computations. On the other hand, for the excited-state computations, the values obtained using the CAM-B3LYP/6-31G(d) functional/basis sets are in better agreement with the experimentally reported values. Therefore, we have calculated the ground states with B3LYP-D3/6-31G(d) and excited states at the CAM-B3LYP/6-31G(d) level of theory. The GaussView 5.0 program package has been used for input files and for visualization of results. Multiwfn 3.8 software has been employed for getting plots of the reduced density gradient (RDG), electron density difference (EDD) and transition density matrix (TDM).

To monitor the performance of the solar cells, the designed dyes have been further investigated after their binding to the TiO_2_ semiconducting surface. The most common phases of TiO_2_ are rutile, anatase, and brookite, with anatase being the most widely studied due to its significance in photocatalysis and surface chemistry. In this study, we have adopted the anatase Ti_5_O_10_ model to represent the TiO_2_ semiconducting surface. We have utilized a variety of acetic acid derivatives and cyanoacrylic acid derivatives as anchoring groups, which covalently bond to the semiconductor surface. These derivatives are ideal for enabling effective adsorption of dye-sensitizers onto the semiconducting surface. The DFT formalism has been employed to model Ti_5_O_10_ at the atomic level, which allowed for accurate simulations of the atomic structure and electronic properties of the Ti_5_O_10_ surface. In this modeling, the positions of the Ti and O atoms have been optimized to replicate realistic surface structures, ensuring that the stoichiometry and morphology align with experimental observations. Each Ti atom is sixfold coordinated, while the O atoms are threefold coordinated. DFT calculations indicate that the anatase (101) surface is the most active, as it contains unpassivated Ti and O atoms, making it widely used for simulating the interface electron transfer process in DSSCs.^37,38^ For the optimization of the dye–TiO_2_ complexes, the split valence approach has been applied. The B3LYP-D3/6-31G(d) level of theory has been employed for atoms other than the Ti atom and for the Ti atom the B3LYP-D3 functional along with the LANL2DZ basis set has been employed.^39^

## 3 Theoretical methodology

The energy required to remove an electron from the neutral species of a molecule is known as the ionization potential (IP). On the other hand, electron affinity (EA) is termed as the difference between the energies of the neutral and anion species of a molecule in their lowest energy states.^40^ The IPs and the EAs of the designed dyes have been calculated by using eqn (1) and (2), respectively:^41,42^

1 IP = *E*^+^(M°) − *E*°(M°),

2 EA = *E*°(M°) − *E*^−^(M°),

where *E*°, *E*^+^ and *E*^−^ are the energies of the dyes in their neutral, cationic and anionic states, respectively, and M° represents the neutral geometry of the dyes.

The reorganization energy (*λ*) refers to the change in energy due to the structural reorganization of the dye molecule to reduce the impact of its excess charge.^39^ There are typically two contributions of *λ*, the outer-sphere and the inner-sphere contribution. The outer-sphere contribution is caused by electron/nucleus relaxation or polarization of the surrounding medium, while the inner-sphere contribution is caused by the geometry relaxation process, which is connected with the charge received or released by the dye molecule. In this work, we have taken into account only the *λ* values contributed by the inner-sphere portion. The *λ* values for cationic species (*λ*_+_) and anionic species (*λ*_−_) can be calculated by using eqn (3) and (4), respectively:^43^

3 *λ*_+_ = [*E*^+^(M°) − *E*°(M°)] − [*E*^+^(M^+^) − *E*°(M^+^)],

4 *λ*_−_ = [*E*°(M^−^) − *E*^−^(M^−^)] − [*E*°(M°) − *E*^−^(M°)].

Here, M^+^ and M^−^ represent the cationic and anionic geometry of the dyes, respectively.

The power conversion efficiency (PCE or *η*) of a photovoltaic device is generally expressed using the following equation:^42^5
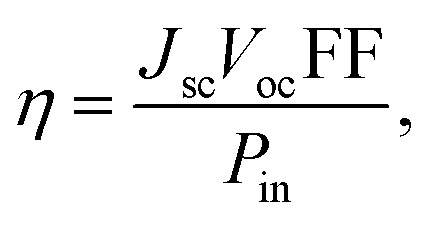
where *J*_sc_, *V*_oc_, *P*_in_ and FF represent the short-circuit photocurrent density, open-circuit voltage, input power of incident sunlight and fill factor, respectively.

The mathematical expression for *J*_sc_ can be defined as^42^:6
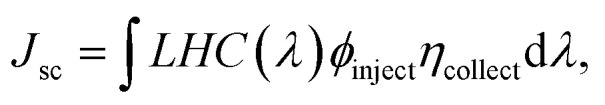
where LHC(*λ*), *ϕ*_inject_ and *η*_collect_ represent the light harvesting capacity at a given wavelength, electron injection efficiency and electron collection efficiency, respectively.^42^

Light harvesting capacity (LHC) is an important parameter that helps in calculating the efficiency of a dye, obtained using the following equation:^42^

7 LHC = 1 − 10^−*f*_osc_^,

where *f*_osc_ represents the oscillator strength corresponding to the wavelength of the absorption maximum.^42^ The excited-state oxidation potential (ESOP) is directly connected to the thermodynamic driving force (Δ*G*^inj^) for the movement of excited electrons from the oxidized dye to the conduction band (CB) of the semiconductor and is represented by eqn (8):^42^

8 Δ*G*^inj^ = ESOP − *E*_CB_.

The calculation of ESOP relative to the CB of TiO_2_ is a key predictive tool for designing of new dyes. For effective electron injection, the ESOP of the dye should be greater than the CB of TiO_2_ (*i.e.*, −4.0 eV). The ESOP values can be estimated by using eqn (9):^42^

9 ESOP = GSOP + *E*_g_.

Here, GSOP (ground-state oxidation potential) represents the difference in energies between the neutral and oxidized charged species and *E*_g_ is the first excitation energy. GSOP can be calculated by using eqn (10):^44^

10 GSOP = *E*°(M°) − *E*^+^(M°).

It is necessary that the GSOP value of the dye lies below the redox potential of the electrolyte I^−^/I_3_^−^ (−4.8 eV).^44^ Several investigations have indicated that the photovoltaic performance of a DSSC is highly dependent on the driving force of regeneration of the dye. Dye regeneration, quantified by Δ*G*^reg^, is a process in which the oxidized dye after electron injection is regenerated by the redox electrolyte. Δ*G*^reg^ can be evaluated by using eqn (11):^39^

11 Δ*G*^reg^ = *E*^redox^(I^−^/I_3_^−^) − GSOP.

The charge transfer rates (*k*_CT_) can be calculated using Marcus theory and are represented by eqn (12):^43^12
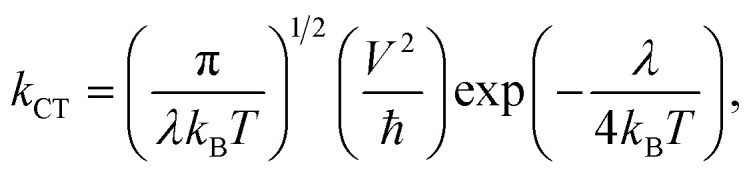
where *k*_B_ is the Boltzmann constant, *T* is the absolute temperature, *V* is the electronic coupling matrix element between two adjacent dyes, and ℏ is the reduced Planck constant. The electronic coupling matrix element for holes (*V*_+_) and electrons (*V*_−_) between two dye molecules can be given by eqn (13):^43^13
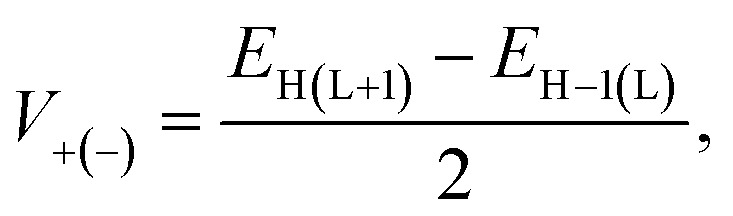
where, *E*_L_, *E*_L+1_, *E*_H_, and *E*_H−1_ represent the energies of the LUMO, LUMO + 1, HOMO, and HOMO − 1, respectively. Hopping mobility (*µ*_hop_), which gives an idea of the quantification of the electron or hole transporting character of the dye molecule, can be given by eqn (14):^43^14
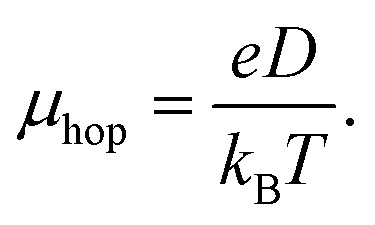
Here, *e* denotes the electronic charge and *D* is the diffusion coefficient. In a one-dimensional charge transfer process, the value of *D* can be calculated by using eqn (15):^43^15
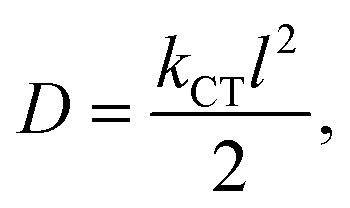
where *l* represents the space distance between two interacting dye molecules.

## 4 Results and discussion

### 4.1 Dihedral angles

**Fig. 2:**
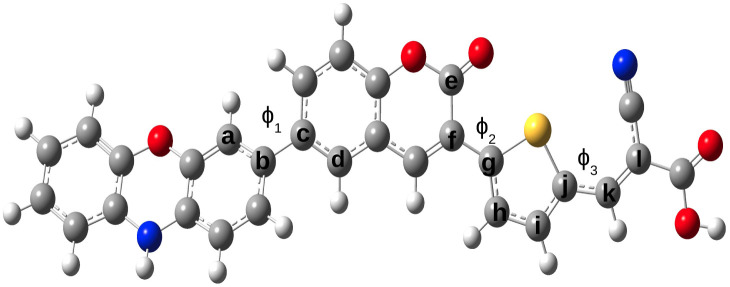
Representation of dihedral angles in a molecule, where *ϕ*_1_ = abcd, *ϕ*_2_ = efgh and *ϕ*_3_ = ijkl.

The optoelectronic characteristics of a molecule are closely related with its ground-state geometry. One of the most essential characteristics demonstrating the ease of charge transfer is the planarity of structures.^45^ A higher dihedral angle value will distort the molecule more and make it less stable. A molecule with a smaller dihedral angle value has less unevenness and is thus more stable. When the dihedral angle is small, the cyanoacrylic group is coplanar with the π-spacer as well as the coumarin and auxiliary donor. Thus, the electron transfer from the donor to the acceptor through the π-conjugated linker is easier, as the dye molecule is coplanar.^46,47^ From the ground-state calculations, we have evaluated the dihedral angles between the auxiliary donor and donor unit (*ϕ*_1_), donor and π-bridge (*ϕ*_2_) and π-bridge and acceptor group (*ϕ*_3_) for all the designed dyes. A pictorial representation of the dihedral angles in a dye has been provided in Fig. 2 and the obtained values have been reported in Table 1.

**Table 1:** The dihedral angle values between distinct portions

Dyes	*ϕ*_1_ (°)	*ϕ*_2_ (°)	*ϕ*_3_ (°)
D1	7.44	7.29	0.68
D2	8.09	9.88	1.12
D3	7.81	9.26	0.94
D4	7.74	8.38	0.84
D5	7.84	9.40	0.95
D6	7.93	9.51	1.09
D7	7.50	8.32	0.79
D8	7.96	9.68	1.06

From Table 1, it has been observed that all the designed dyes are almost planar in structure since the dihedral angles between their D–D, D–π and π–A moieties are effectively small (below 10°). The dihedral angles between the auxiliary donor and donor unit (*ϕ*_1_) are in the range of 8° to 9°, for the donor and π unit (*ϕ*_2_) are in the range of 7° to 10° and for the π and acceptor unit (*ϕ*_3_) are in the range of 0*°* to 2*°* for all the dyes. Considering the dihedral angles between all the fragments are less than 10°, this indicates that each auxiliary donor along with the primary coumarin donor has its π-conjugated thiophene bridge located coplanar with the cyanoacrylic acid group, acting as both the acceptor and the anchoring group on the TiO_2_ surface. These coplanar structures between the donor, linker and acceptor led to a strong conjugation effect as well as allowing effective injection into the conduction band of TiO_2_ through the cyanoacrylic acid group. Among the studied dyes, dye D1, having a phenoxazine auxiliary donor, possesses the lowest values of dihedral angle among all the fragments. Consequently, dye D1 possesses the most planar structure among all the designed dyes. Analyzing their structural properties, we can conclude that incorporation of an efficient auxiliary donor can enhance the extent of electron delocalization of the sensitizer, which in turn demonstrates the ease of charge transfer.

### 4.2 Frontier molecular orbital analysis

The efficiency of DSSCs is known to be significantly influenced by the electronic characteristics of the dye. These parameters include the highest occupied molecular orbital (HOMO) and lowest unoccupied molecular orbital (LUMO) energy levels and their energy gap (*Δ*_H–L_). HOMO and LUMO contributions are very important in determining the charge-separated states of dye sensitizers. *Δ*_H–L_ is defined as the difference between the LUMO and HOMO energies. In general, a smaller energy gap of the dye results in ease of transporting electrons from the HOMO to LUMO energy levels through absorption of light energy with an appropriate wavelength. Intramolecular charge transfer increases when the energy gap between the HOMO and LUMO decreases. The HOMO and LUMO energies have been calculated, and their values are presented in Table 2, along with the *Δ*_H–L_ values.

**Table 2:** HOMO and LUMO energies and their corresponding *Δ*_H–L_ values

Dyes	HOMO (eV)	LUMO (eV)	*Δ*_H–L_ (eV)
D1	−4.92	−2.88	2.04
D2	−5.22	−2.95	2.27
D3	−5.04	−2.92	2.12
D4	−4.93	−2.84	2.09
D5	−5.05	−2.88	2.17
D6	−5.06	−2.81	2.25
D7	−4.93	−2.86	2.07
D8	−5.10	−2.90	2.20

Table 2 shows that the studied dyes have *Δ*_H–L_ values ranging from 2.04 eV to 2.27 eV. This range falls within the reported 1 eV to 4 eV range needed for semiconducting devices.^48^ This suggests that our designed dyes are favourable for DSSCs. The calculated *Δ*_H–L_ values are in the following order: D2 > D6 > D8 > D5 > D3 > D4 > D7 > D1. It is evident that dye D1 exhibits the lowest *Δ*_H–L_ value (2.04 eV), while dye D2 shows the highest (2.27 eV). Compared to the triphenylamine-based donors (D7 and D4), the phenoxazine donor in dye D1 demonstrates a stronger electron-donating capability. This is attributed to the presence of electron-rich oxygen and nitrogen atoms within its heterocyclic ring, which significantly enhance its electron-donating ability.^49^ However, increasing the number of methoxy groups on the triphenylamine unit enhances its electron-donating ability, thereby enabling fine-tuning of the target properties. The stronger π-delocalization facilitated by the cyclopentadithiophene and benzothiadiazole moieties further boosts the donor strength of the indoline units (as seen in D3 and D5). Consequently, these compounds demonstrate potential as promising candidates for photovoltaic applications.

**Fig. 3:**
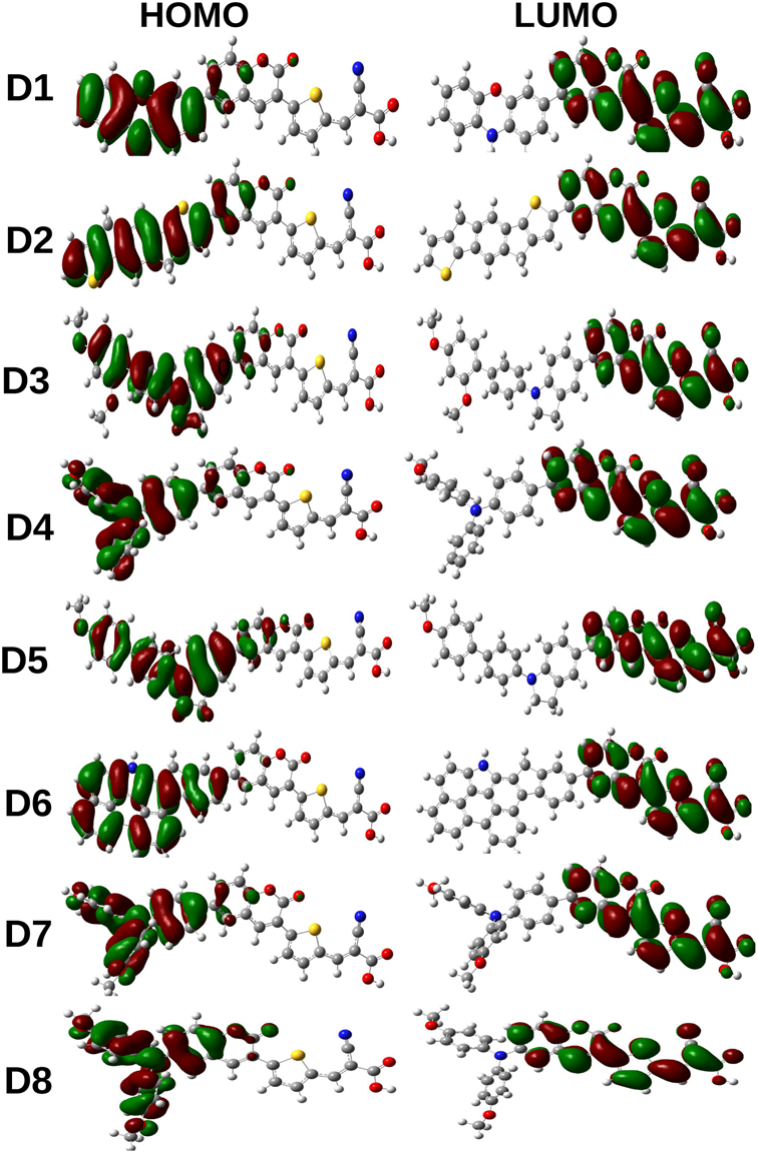
Plot of frontier molecular orbitals of the designed dyes.

As frontier molecular orbital (FMO) analysis is an effective approach for exploring the optoelectronic characteristics, we have represented the FMO diagrams for all the dye systems in Fig. 3. From Fig. 3, it is observed that the HOMOs are delocalized over the coumarin and auxiliary donor parts. On the other hand, the LUMOs are delocalized over the acceptor part, with some contribution from the π-bridge unit. This distribution pattern of HOMO and LUMO over the donor, acceptor and linker clearly demonstrates the occurrence of charge separation in our designed dyes and demands valuable consideration for future applications.

### 4.3 Reduced density gradient

**Fig. 4:**
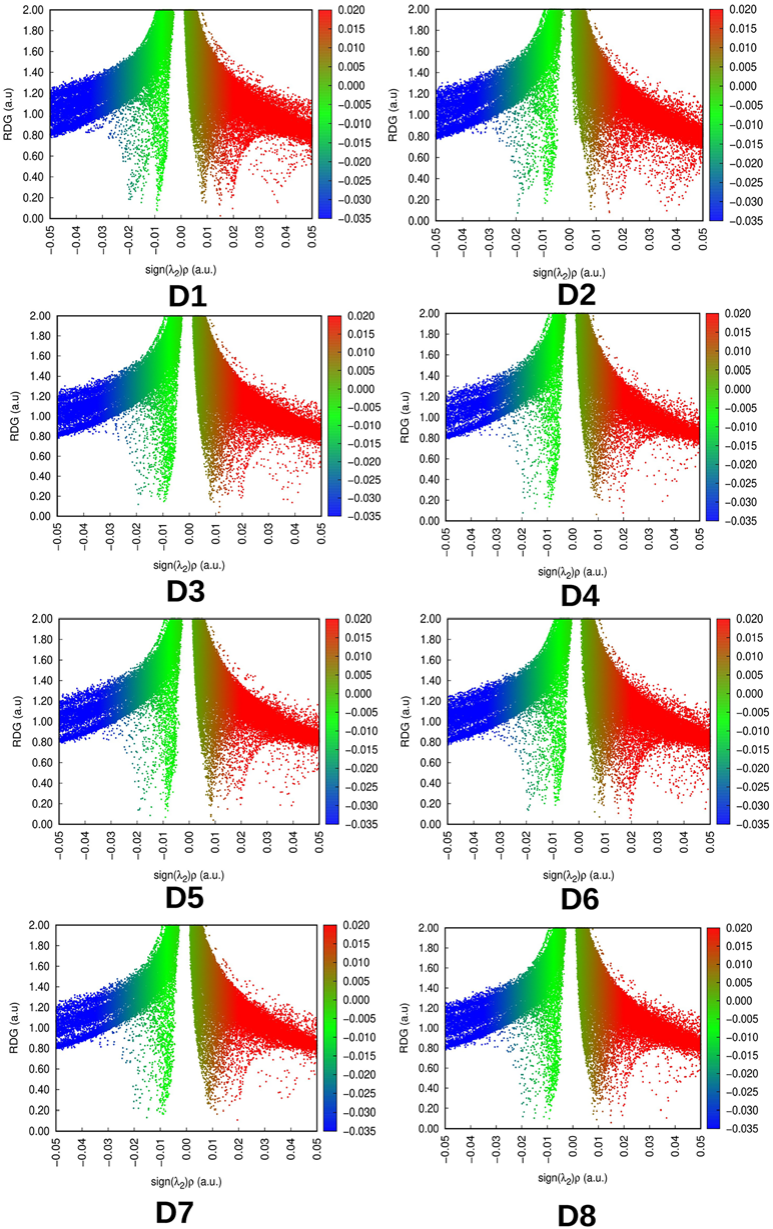
Plot of reduced density gradient of the designed dyes.

Examining the reduced density gradient (RDG) of dyes provides insight into non-covalent interactions within molecules. The RDG graph, a two-dimensional representation, illustrates various interactions occurring within the molecule, including van der Waals interactions, steric interactions, and hydrogen bonding.^50^ To comprehensively grasp the intensity and nature of these non-covalent interactions, RDG graphs have been generated using Multiwfn 3.8 software for all the designed dyes. The characterization of each interaction is determined by *λ*_2_ in the non-covalent interaction analysis. A lower value of *λ*_2_ indicates the presence of significantly less forces of attraction present in that part of molecule. When the *λ*_2_ value rises above zero, it indicates the existence of repulsive forces. Less repulsive and more attractive interactions will increase the stability of molecules, and strong H-bonding will also lead to better stability.^51,52^ The RDG plots have been displayed in Fig. 4. Fig. 4 highlights the prevalence of attractive forces, particularly van der Waals forces, over steric forces, affirming the stability of the designed dyes. The blue segment, situated on the left side of each plot, is positioned higher than the red segment on the right, indicating the predominance of attractive forces over repulsive forces in all the designed dyes. This illustrates the stability of these molecules.

### 4.4 Natural bond orbital

As this work concentrates on identifying the most effective auxiliary donor moiety among the eight dyes, natural bond orbital (NBO) analysis is pivotal in selecting the appropriate component for a dye molecule. A NBO analysis has been carried out on the optimized structure to comprehend the charge distribution and electron transfer mechanisms of the dyes.^53^ It can be used to measure the extent of charge transfer occurring in the ground state.^54^ The calculated NBO charges for the donor, π-bridge, and acceptor are presented in Table 3. The calculated NBO values show that the donor component in all dyes carries a positive charge, suggesting its effectiveness as an electron donor unit. However, the NBO charges of the π bridge and acceptor possess a negative charge across all dyes. The positive and negative NBO values reflect the capacity of a specific unit to donate and accept electrons, respectively. The greater the positive charge present on the donor moiety, the stronger its indication of being the most efficient electron donor. Conversely, a higher negative charge on the acceptor part signifies a more effective electron acceptor unit.^53^ This analysis reveals that among all the dyes, phenoxazine along with a coumarin donor in dye D1 demonstrated the maximum positive NBO charge, indicating its potential to serve as the most effective donor component of the dye. The higher NBO values observed for D1 and D7 (dimethoxy-substituted triphenylamine with a coumarin donor unit) indicate that their respective dyes have a significant capacity to donate electrons from the donor to the acceptor *via* the linker, facilitating easy adsorption onto the TiO_2_ cluster. Based on the findings above, it is evident that both the phenothiazene and trimethylamine units serve as promising auxiliary donating groups to enhance the photovoltaic properties of dye sensitizers. The NBO analysis further verifies the emergence of a charge-separated state within the dye molecule, substantiating the occurrence of intramolecular charge transfer between donor and acceptor moieties.

**Table 3:** NBO values of the studied dyes

Dyes	Donor	π-bridge	Acceptor
D1	0.333	−0.112	−0.221
D2	0.286	−0.107	−0.179
D3	0.311	−0.104	−0.207
D4	0.313	−0.101	−0.212
D5	0.303	−0.103	−0.200
D6	0.291	−0.105	−0.186
D7	0.330	−0.112	−0.218
D8	0.299	−0.102	−0.197

### 4.5 Oxidation potential of the dyes

**Fig. 5:**
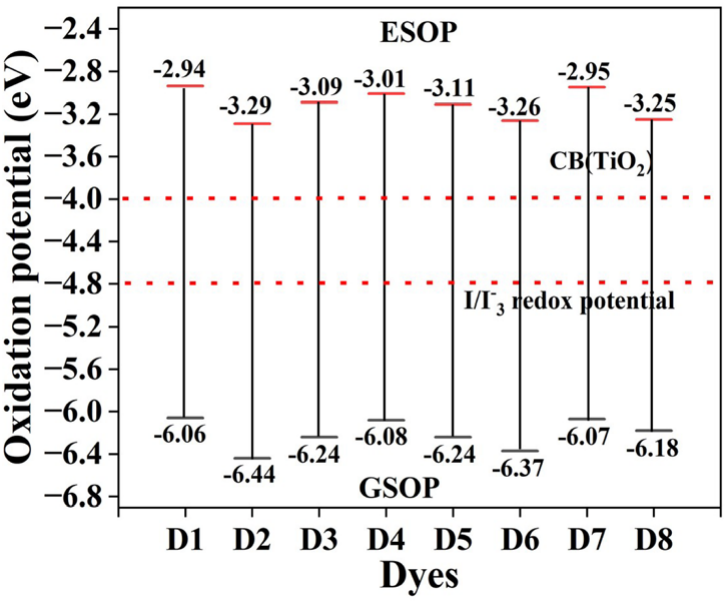
Plot of band alignment of the designed dyes with respect to the CB of TiO_2_ and the redox potential of I^−^/I_3_^−^.

It is well known that for efficient electron injection and dye regeneration processes, the frontier orbitals of the dye and semiconductor need to be properly aligned.^55^ We have determined the GSOP and ESOP values for the designed dyes in order to investigate the possibility of spontaneous electron injection and dye regeneration processes. For a spontaneous dye regeneration process, the GSOP values of the dyes must lie below the redox potential of the I^−^/I_3_^−^ electrolyte. The redox potential for the I^−^/I_3_^−^ couple is found to be −4.8 eV.^56^ Similarly, in order to initiate spontaneous electron injection, the ESOP values must lie above the conduction band of the TiO_2_ semiconducting surface (*i.e.*, −4.0 eV).^39,56^ Additionally, we computed the Δ*G*^reg^ and Δ*G*^inj^ values to gain insight into the charge transfer characteristics. The Δ*G*^inj^, ESOP, GSOP, and Δ*G*^reg^ can be calculated by using eqn (8)–(11), respectively. Here, we adopt the Ti_5_O_10_-cluster as the semiconductor surface due to its high photostability and effective charge separation, with a low cost of production.^57^ The calculated values of GSOP, ESOP, Δ*G*^reg^ and Δ*G*^inj^ have been reported in Table 4. We have also presented a plot of the band alignment of the dyes with respect to the CB of the TiO_2_ semiconducting surface and the redox potential of the I^−^/I_3_^−^ electrolyte in Fig. 5.

**Table 4:** GSOP, ESOP, Δ*G*^reg^ and Δ*G*^inj^ values (in eV units)

Dyes	GSOP	ESOP	Δ*G*^reg^	Δ*G*^inj^
D1	−6.06	−2.94	1.26	1.05
D2	−6.44	−3.29	1.64	0.70
D3	−6.24	−3.09	1.44	0.90
D4	−6.08	−3.01	1.28	0.98
D5	−6.24	−3.11	1.44	0.88
D6	−6.37	−3.26	1.57	0.73
D7	−6.07	−2.95	1.27	1.04
D8	−6.18	−3.25	1.38	0.74

From Table 4 and Fig. 5, it has been observed that the GSOP values of the dyes lie below the redox potential of the I^−^/I_3_^−^ electrolyte couple and the ESOP values lie above the conduction band of the TiO_2_. From this observation, it is obvious that our designed dyes exhibit spontaneous electron injection from the excited state of the dyes to the conduction band of the TiO_2_ semiconducting surface and thus spontaneous regeneration of dyes from the electrolyte couple. This demonstrates that all our designed dyes have the ability to serve as potential candidates for DSSC fabrication.

The estimation of the rate of electron injection (Δ*G*^inj^) and dye regeneration (Δ*G*^reg^) is very important for studying photovoltaic characteristics. It is notable that higher Δ*G*^inj^ values of the dyes indicate higher efficiency towards electron injection.^42,56^ As shown in Table 4, the values of Δ*G*^inj^ for all the dyes are in the range of 0.70 to 1.05 eV, implying that the electrons of the dyes have been effectively injected from the anchoring group into the conduction band of the TiO_2_ semiconductor surface under photoexcitation. Also, according to Table 4, the Δ*G*^reg^ values of all the designed dyes are positive. This indicates that the redox level of the electrolyte is higher than the ground state of the dyes and hence responsible for the suppression of electron recombination. For the dye to regenerate more quickly and efficiently, the Δ*G*^reg^ value has to be low.^58^ Here, all computed Δ*G*^reg^ values are between 1.26–1.64 eV. In this regard, we can conclude that the studied dyes are promising towards dye regeneration and electron injection.

The ionization potential (IP) and electron affinity (EA) are two exemplifying criteria for demonstrating the charge transfer characteristics. The amount of energy needed for ionization of a molecule is termed as IP, which is the energy difference between the cationic and neutral states. Conversely, EA is the ability to make anions by accepting electrons, which is the difference in energy at the ground states between neutral and anionic molecules.^56^ Both of these parameters can be calculated using eqn (1) and (2), respectively, and the corresponding values are given in Table 5. It is already well known that a low IP speeds up the creation of holes by facilitating the removal of electrons. Furthermore, a high value of EA indicates that it will be challenging to remove the conduction-band electrons. The EA value of the dye molecule explains the recombination between the injected electron and the oxidized dye species. A lower EA value ensures easy removal of electrons from the conduction band. Therefore, in order to transport electrons to the semiconducting surface effectively, a dye sensitizer has to have a low EA value.^29^Table 5 illustrates that IP is found to be between 6.06 and 6.44 eV for all the designed dyes. This suggests that the dyes have superior stability against oxidation. Conversely, the projected range for the EA values is 1.59–1.85 eV. This indicates that our designed dyes meet the requirements for becoming a suitable dye sensitizer.

**Table 5:** Calculated values of IP and EA (in eV units)

Dyes	IP	EA
D1	6.06	1.59
D2	6.44	1.85
D3	6.24	1.63
D4	6.08	1.60
D5	6.24	1.64
D6	6.37	1.72
D7	6.07	1.59
D8	6.18	1.70

### 4.6 Absorption properties

Solar cells convert sunlight into electricity through the photovoltaic effect. The absorption properties play a significant role in determining how effectively a solar cell can capture and convert sunlight into electrical energy.^56^ To gain a deeper understanding of the absorption properties of the designed dyes, the excitation properties have been calculated for 30 excited states utilizing the CAM-B3LYP/6-31G(d) level of theory. We have calculated the dipole moment (*µ*), excitation energy (*E*_g_), maximum absorption wavelength (*λ*_max_), light harvesting capacity (LHC) and oscillator strength (*f*_osc_), and reported them in Table 6. For an efficient DSSC, the sensitizer must possess a high oscillator strength and a low HOMO–LUMO gap, and it must absorb in a broad range of the UV-visible spectrum.^30^

**Table 6:** Calculated *E*_g_, *λ*_max_, *f*_osc_, electronic transitions, LHC and *µ*

Dyes	*E*_g_ (eV)	*λ*_max_ (nm)	*f*_osc_	Transitions	LHC	*µ* (Debye)
D1	2.02	522	2.04	H → L (83%)	0.990	13.23
D2	3.15	400	1.26	H → L (61%)	0.945	10.92
D3	2.37	481	1.79	H → L (70%)	0.983	12.46
D4	2.21	498	1.93	H → L (73%)	0.988	12.76
D5	2.52	470	1.67	H → L (68%)	0.978	12.20
D6	2.97	420	1.49	H → L (65%)	0.967	11.05
D7	2.09	506	2.01	H → L (80%)	0.990	13.01
D8	2.71	434	1.55	H → L (67%)	0.971	11.46

**Fig. 6:**
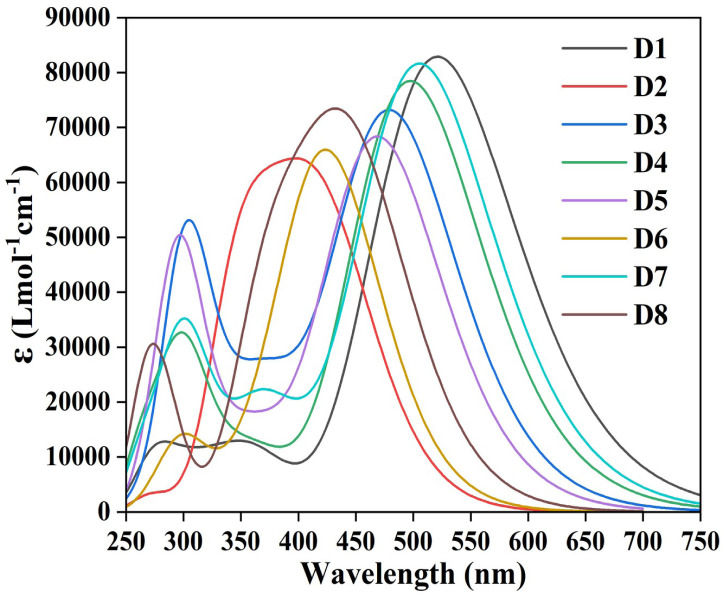
UV-visible spectra of the designed dyes.

Specifically, *f*_osc_ represents the probability of electromagnetic radiation absorption during transition between the energy levels of a particular molecule.^59^ From Table 6, it has been observed that all the designed dyes possess comparatively high *f*_osc_ values. Among all the designed dyes, the D1 dye has the highest absorption maximum (522 nm) with maximum oscillator strength (2.04). This may be due to the (+I) effect of the phenoxazine donor group attached to the D1 dye. The presence of strong electron-donating and strong electron-accepting groups can reduce the HOMO–LUMO gap, thereby facilitating efficient electron transfer from the donor to the acceptor group. This can result in a redshift of the absorption maximum and an increase in oscillator strength. Thus, the high oscillator frequency and high absorption maxima exhibited by all designed dyes may be due to the +I effect of the donor moieties at the peripheral positions. Furthermore, it is evident that the electronic transitions with the highest wavelength of absorption occur primarily due to the H → L transitions (61–83%) for all the dyes. The corresponding UV-visible spectra for the designed dyes have been provided in Fig. 6.

The dipole moment (*µ*) is a measure of the separation of positive and negative charges within a molecule that gives information about its polarity. Higher values of *µ* indicate that the designed dyes are polar in nature.^56^ In donor–acceptor systems, where a dye is designed to donate electrons from donor to acceptor moieties, a higher dipole moment can enhance the separation of charges upon absorption of light. From Tables 6, it has been observed that all the designed dyes possess comparatively high values of *µ*. Among them, D1 and D7 have higher values of *µ* due to the phenoxazine and dimethoxy-substituted triphenylamine donor groups.

We have calculated LHC to predict the *J*_sc_ values using eqn (6). From eqn (6), it is evident that a high LHC value will lead to a high *J*_sc_ value. Table 6 shows that all the dyes have relatively high LHC values, ranging from 0.945 to 0.990. Furthermore, D1 and D7 possess the highest LHC values (*i.e.*, 0.990) among all studied dyes. Based on the LHC values, D1 and D7 are expected to have the greatest *J*_sc_ values among all the designed dyes, which can lead to maximum *η*.

The reorganization energy (*λ*) is another crucial factor that influences the efficiency of DSSCs. The energy cost due to the conformational change during photoexcitation is defined as the reorganization energy.^39^ Marcus electron transfer theory relates the total reorganization energy to the rate of the electron transfer, which is expressed by eqn (12). We have calculated the reorganization energy for both holes (*λ*_+_) and electrons (*λ*_−_) to understand the charge transportation in the designed dyes and their respective values have been reported in Table 7. For effective charge transportation, the *λ* value (*λ*_+_ or *λ*_−_) needs to be low. Thus, it is necessary to have a low value of *λ* for a high charge transfer rate. A lower *λ*_+_ value reflects the hole transporting nature of the dyes and conversely, a lower *λ*_−_ value reflects the electron transporting nature of the dyes.

**Table 7:** Calculated values of reorganization energies (in eV units)

Dyes	*λ*_+_	*λ*_−_	*λ*_tot_
D1	0.059	0.164	0.223
D2	0.250	0.356	0.606
D3	0.196	0.249	0.445
D4	0.126	0.217	0.343
D5	0.226	0.288	0.514
D6	0.243	0.318	0.561
D7	0.068	0.176	0.244
D8	0.239	0.316	0.555

From Table 7, it has been observed that for all the designed dyes, the *λ*_−_ values are higher than the *λ*_+_ values. As a result, all of our designed dyes behave as hole transporting materials. Additionally, it is noted that, among all the designed dyes, dyes D1 and D7 possess lower *λ*_+_ values compared to the rest. As a result, among all designed dyes, D1 and D7 are expected to have facile hole transportation.

We have also calculated the total reorganization energy (*λ*_tot_) values (*i.e.*, the sum of the values of *λ*_+_ and *λ*_−_) and these are reported in Table 7. For achieving greater electron injection, *i.e.*, to obtain higher current density, the *λ*_tot_ value must be smaller. This will reduce recombination.^42^ From Table 7, it is observed that the designed D1 and D7 dyes possess lower values of *λ*_tot_ compared to the other dyes. This reveals better electron–hole separation efficiency of the D1 and D7 dyes, which may have slower recombination processes compared to the other designed dyes.

**Fig. 7:**
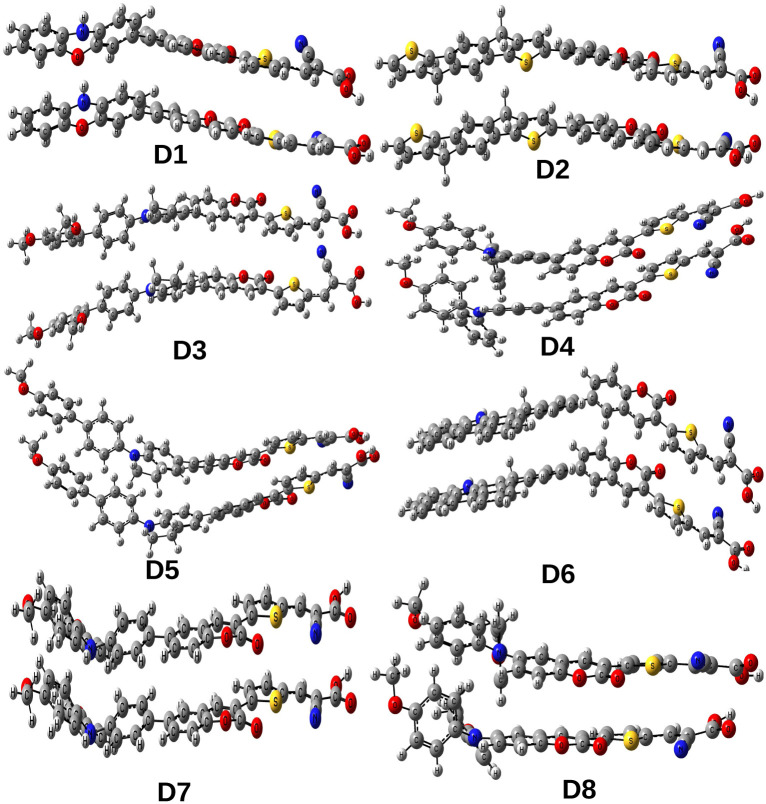
Optimized structures of stacked dyes.

To gauge the electronic coupling matrix element (*V*), we have considered the π-stacking in a cofacial arrangement of the two adjacent dyes. The π-stacking orientation of the dyes has been evaluated by arranging two adjacent dyes in a face-to-face manner at a distance of 3.5 Å. The representative arrangement of two stacked dyes (for all the dye systems) has been presented in Fig. 7 and the calculated *V* values (using eqn (13)) are reported in Table 8. Using these *V* values, we have calculated the charge transfer rates for holes (*k*_CT_^+^) and electrons (*k*_CT_^−^) (using eqn (12)) and reported these in Table 8.

**Table 8:** Calculated values of *V*, *k*_CT_ and *µ*_hop_

Dyes	*V*_+_ (eV)	*V*_−_ (eV)	*k*_CT_^+^ × 10^13^ (s^−1^)	*k*_CT_^−^ × 10^13^ (s^−1^)	*µ*_hop_^+^ (cm^2^ V^−1^ s^−1^)	*µ*_hop_^−^ (cm^2^ V^−1^ s^−1^)
D1	0.032	0.029	47.26	3.178	6.656	4.721
D2	0.012	0.011	12.06	1.103	0.251	0.069
D3	0.257	0.151	18.13	8.116	3.964	0.411
D4	0.262	0.155	20.48	2.152	4.318	0.432
D5	0.077	0.029	17.53	5.994	0.373	1.265
D6	0.071	0.053	10.48	0.169	0.296	0.043
D7	0.048	0.016	46.56	1.712	5.974	0.355
D8	0.082	0.077	16.66	3.495	0.323	0.474

From Table 8, it is evident that the *k*_CT_+ values of the compounds are higher than their corresponding *k*_CT_^−^ values. This result is in accordance with the reorganization energy values. Moreover, it is also apparent from Table 8 that dye D1 and D7 exhibit comparatively higher *k*_CT_^+^ values due to their lower value of *λ*_+_ among all the designed dyes. Another significant measure is the hopping mobility (*µ*_hop_), which helps in determining the conducting capacity of the organic dyes. A high *µ*_hop_ value signifies higher electronic coupling between the adjacent dyes, which in turn indicates a better conducting capacity of the organic dyes. The calculated *µ*_hop_ values for holes (*µ*_hop_^+^) and electrons (*µ*_hop_^−^) are reported in Table 8. From this table, it is observed that among all the studied dyes, D1 and D7 possess higher values of *µ*_hop_^+^. These values are consistent with the observed *k*_CT_^+^ values for these dyes. Therefore, we are hopeful that our designed dyes may serve as potential candidates for the fabrication of optoelectronic devices in the near future.

### 4.7 Electron density difference analysis

**Fig. 8:**
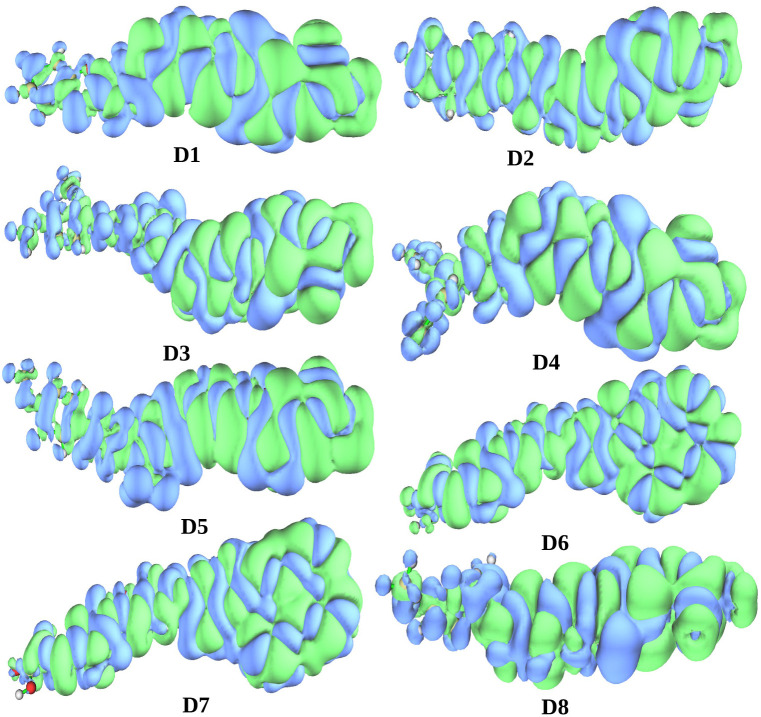
Plot of the electron density differences of all dye systems.

To understand the nature of charge separation in the dyes after electron excitation, we conducted a systematic analysis of their electronic structures.^60^ Using the Multiwfn package, we determined the electron density difference (EDD) map for the dyes. The EDD maps for all the dyes are presented in Fig. 8. In this diagram, the green and blue areas indicate the increase and decrease in electron density resulting from electron excitation. During the S0 → S1 transition, it is evident from Fig. 8 that there has been a transfer of charge from the donor unit to the acceptor unit. Therefore, in all the designed dyes, the donor unit shows the lowest electron density, while the acceptor unit demonstrates the highest electron density. Hence, it can be concluded from the EDD maps that during the transition from S0 → S1, charge transfer occurs from the donor unit to the acceptor unit in all of the designed dyes.

### 4.8 Transition density matrix analysis

**Fig. 9:**
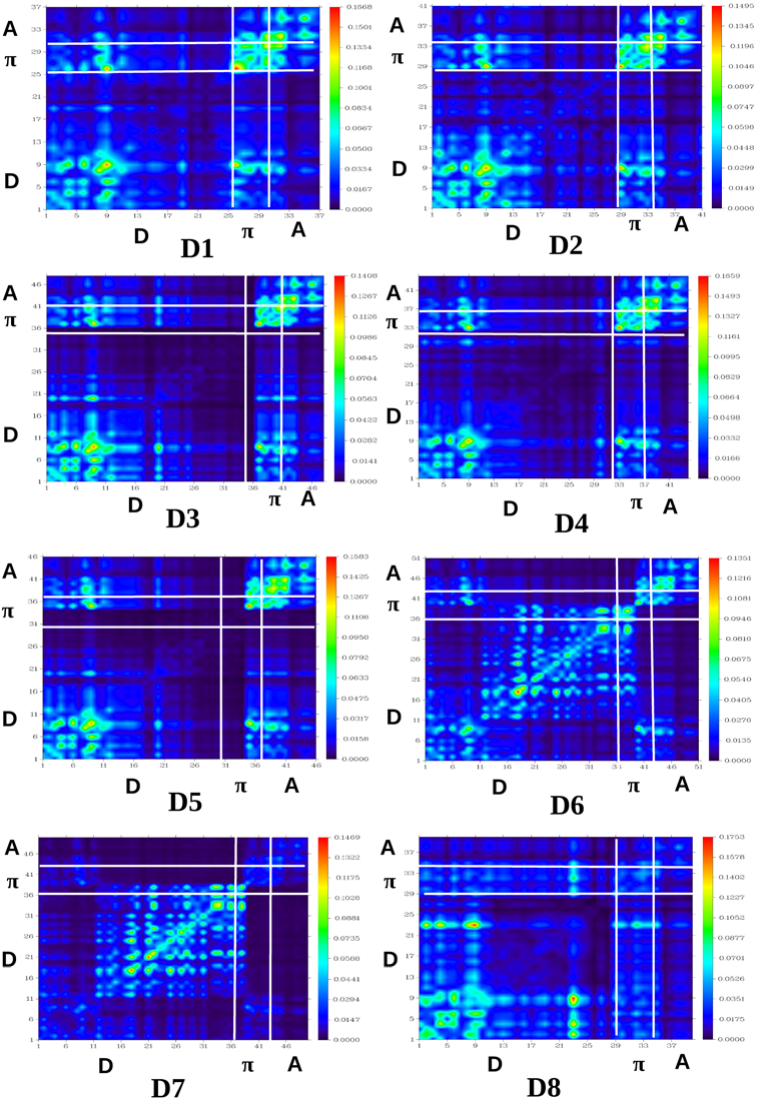
Plots of the transition density matrices of all the dye systems.

In molecular systems, the transition density matrix (TDM) characterizes electronic excitation processes in light-harvesting systems. It uses a spatial map to illustrate the movement of electron density from donor to acceptor (electron–hole pairs) through π-conjugation within the molecules.^61,62^ The *x*-axis and left *y*-axis are labelled with atom numbers, while the right *y*-axis is labelled with coefficients and a color range (transition density increases from blue to red). The phenomenon of charge transfer can be analyzed in relation to the off-diagonal positions, while locally excited states can be observed along the diagonals of the plots.^63^ In the TDM plots, the electronic cloud is represented by different colors indicating its distribution across various regions of the molecule. The brighter regions in the matrix indicate areas with higher population for charge transition.^64^ To gain a more profound insight into the movement of electronic charge density within the dyes, each dye has been divided into three segments, comprising the donor (D), acceptor (A), and π-bridge (π). Atoms with varying fragments are arranged along the bottom and left sides. Atoms are numbered sequentially from 1 to the total number present in the molecule. Here, hydrogen atoms are typically disregarded by default due to their minimal impact on the transition. The TDM plots for all the dyes are presented in Fig. 9. From the TDM analysis, it is evident that all the designed dyes exhibit a consistent distribution of electrons and holes throughout the dyes, as indicated by bright areas diagonally across the figure. All molecules investigated have shown both diagonal and off-diagonal characteristics, which contribute to efficient charge separation. Charge transfer occurs from the core to acceptor regions *via* the π-bridge, indicated by brighter spots in the plots. These continuous bright spots between donor and π-bridge, and π-bridge and acceptor regions in off-diagonal positions, demonstrate effective charge transfer from the donor to the acceptor region. The TDM analysis shows that the D1, D2, and D7 molecules have a uniform electron distribution throughout the molecule, moving diagonally as indicated by the bright areas in the figure. Although the other dyes also show uniform movement of charge density, it is less pronounced than in the D1, D2, and D7 dyes. Thus, the TDM heat maps show that electrical charges are successfully transferred from the donor to the acceptor without being trapped by charge coherence.

### 4.9 Adsorption on the TiO_2_ surface

**Fig. 10:**
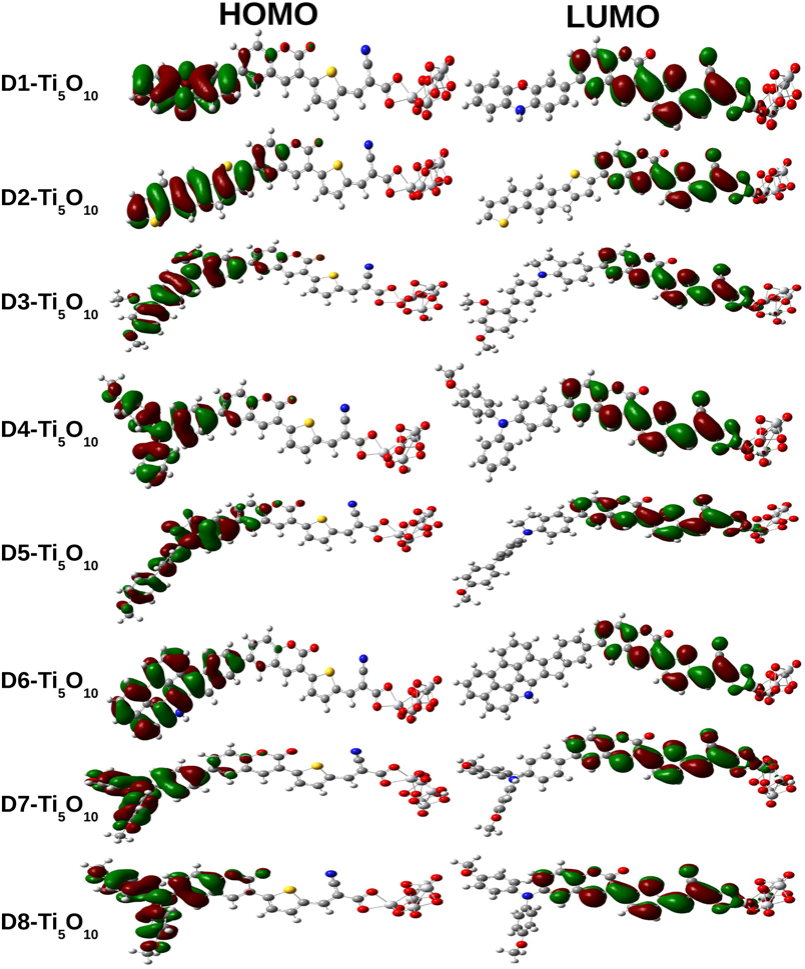
Frontier molecular orbitals of dye–Ti_5_O_10_ clusters.

It is extremely important to investigate the properties of the dyes along with the surface of TiO_2_ in order to observe the realistic performance of a solar cell device. In this work, we have adopted a Ti_5_O_10_ cluster to represent the TiO_2_ semiconductor surface. The justification for choosing the Ti_5_O_10_ cluster is provided in the SI (Table S3 and Fig. S2). Here, we have used cyanoacrylic acid as the anchoring group to enable the adsorption of the dye-sensitizers to the semiconducting surface. This anchoring group has been proven to be the most ideal anchoring group in DSSCs.^42^ We have represented the FMO diagrams of the designed dye–Ti_5_O_10_ clusters in Fig. 10.

As demonstrated in Fig. 10, the HOMOs in all the dye clusters are mainly delocalized over the donor portion of the dyes. Conversely, the LUMOs are primarily delocalized over the acceptor portion with some contributions from the π-bridging unit as well as the Ti_5_O_10_ cluster. Thus, this analysis demonstrates the existence of an efficient charge transfer mechanism in the dye–Ti_5_O_10_ clusters.

**Fig. 11:**
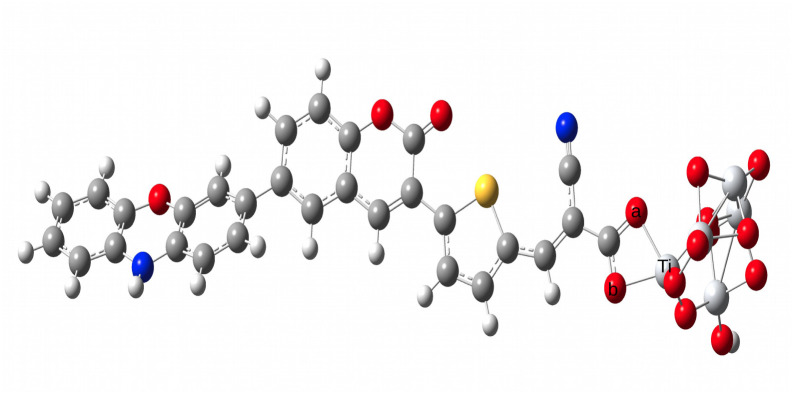
Representation of the Ti–O bond lengths in the dye–Ti_5_O_10_ cluster.

As a part of our calculations, we have also calculated the Ti–O bond lengths of the designed dyes and the results are presented in Table 9. The representation of the Ti–O bond length in the representative D1–Ti_5_O_10_ cluster is presented in Fig. 11. Table 9 shows that all the designed dyes have Ti–O bond lengths ranging from 2.034–2.049 Å. These values are almost in agreement with the theoretically reported Ti–O bond lengths (2.03–2.24 Å) for various dye–TiO_2_ clusters.^42^ These observations suggest that all the designed dyes undergo chemisorption on the TiO_2_ surface.

**Table 9:** Ti–O bond lengths of the dye–Ti_5_O_10_ clusters

Dye–Ti_5_O_10_	Ti–O_*a*_ (Å)	Ti–O_*b*_ (Å)
D1–Ti_5_O_10_	2.034	2.046
D2–Ti_5_O_10_	2.037	2.040
D3–Ti_5_O_10_	2.035	2.049
D4–Ti_5_O_10_	2.036	2.040
D5–Ti_5_O_10_	2.035	2.049
D6–Ti_5_O_10_	2.036	2.040
D7–Ti_5_O_10_	2.037	2.040
D8–Ti_5_O_10_	2.036	2.049

Additionally, we have computed the *Δ*_H–L_ values and ground-state dipole moments (*µ*_g_) for all the designed dye–Ti_5_O_10_ clusters. The results have been reported in Table 10.

**Table 10:** Calculated *Δ*_H–L_ and *µ*_g_ values of the dye–Ti_5_O_10_ clusters

Dye–Ti_5_O_10_	*Δ*_H–L_ (eV)	*µ*_g_ (Debye)
D1–Ti_5_O_10_	1.57	20.41
D2–Ti_5_O_10_	1.86	15.06
D3–Ti_5_O_10_	1.66	18.06
D4–Ti_5_O_10_	1.60	18.56
D5–Ti_5_O_10_	1.74	17.55
D6–Ti_5_O_10_	1.85	16.05
D7–Ti_5_O_10_	1.58	19.18
D8–Ti_5_O_10_	1.80	16.75

From the comparison of Tables 2 and 10, it has been observed that the *Δ*_H–L_ values of the dye–Ti_5_O_10_ clusters are lower than those of the isolated dyes. Moreover, from the comparison of Tables 6 and 10, it has been observed that the dipole-moment values of the dye–Ti_5_O_10_ clusters (*µ*_g_) are higher than those of the isolated dyes (*µ*). These observations indicate that binding of the dyes to the Ti_5_O_10_ semiconducting surface leads to the enhancement of their charge transport characteristics.

**Fig. 12:**
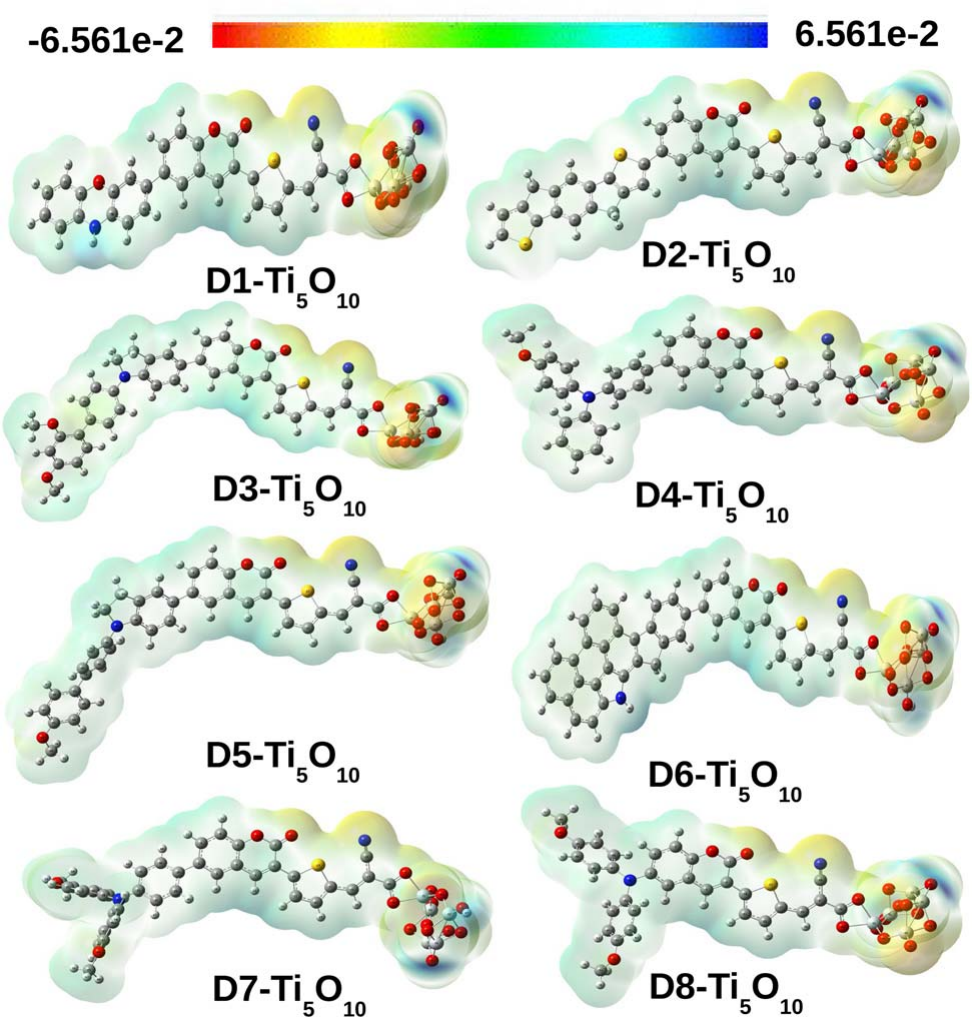
MEPS contour plots for the designed dye–Ti_5_O_10_ clusters.

The molecular electrostatic potential surface (MEPS) is a popular way of visualizing the electrostatic nature of dye molecules. The MEPS serves as a useful resource, offering qualitative insights into charge transportation from the donor moiety to the acceptor moiety through the π-linker.^65^ The positive potential increases in the following color order: red < orange < yellow < green < blue. Here, the blue and red colors depict the electron-deficient and electron-rich regions, respectively. We have presented the MEPS plots of the designed dye–Ti_5_O_10_ clusters in Fig. 12.

From Fig. 12, it is observed that for all designed dye–Ti_5_O_10_ clusters, the positive charge is spread over the dyes and the negative charge is spread over the Ti_5_O_10_ surface. This observation signifies the charge transfer characteristics of all the designed dyes.

In order to study the absorption properties of the dye–Ti_5_O_10_ clusters, we have computed the excitation energies (*E*_g_), maximum absorption wavelengths (*λ*_max_), oscillator strengths (*f*_osc_), transitions, and contributions of the frontier orbitals. The results are shown in Table 11.

**Table 11:** *E*_g_, *λ*_max_, *f*_osc_, transitions and *E*_b_ values of the dye–Ti_5_O_10_ clusters

Dyes	*E*_g_ (eV)	*λ*_max_ (nm)	*f*_osc_	Transitions	*E*_b_ (eV)
D1–Ti_5_O_10_	2.15	536	2.10	H → L (90%)	0.20
D2–Ti_5_O_10_	3.21	465	1.63	H → L (68%)	0.30
D3–Ti_5_O_10_	2.42	515	1.90	H → L (73%)	0.26
D4–Ti_5_O_10_	2.37	520	2.01	H → L (78%)	0.28
D5–Ti_5_O_10_	2.60	507	1.82	H → L (70%)	0.29
D6–Ti_5_O_10_	2.99	470	1.71	H → L (69%)	0.23
D7–Ti_5_O_10_	2.18	530	2.06	H → L (87%)	0.22
D8–Ti_5_O_10_	2.84	460	1.76	H → L (71%)	0.28

**Fig. 13:**
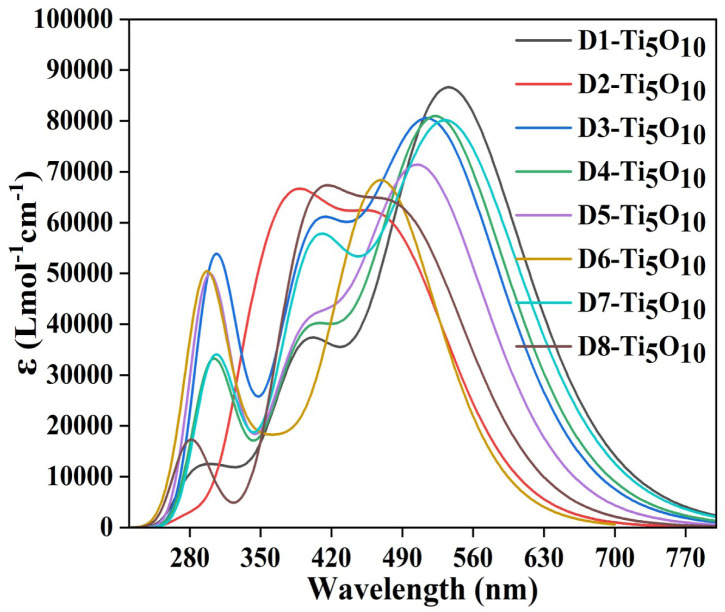
The plots of the UV-visible spectra of all the designed dye–Ti_5_O_10_ clusters.

Table 11 illustrates that the dye–Ti_5_O_10_ clusters experienced higher maximum absorption wavelength (*λ*_max_) values and lower excitation energies (*E*_g_). This finding suggests that the adsorbed dyes have a red shift as compared to the isolated dyes and follow the same trend. The absorption maxima for all the dye–Ti_5_O_10_ clusters are primarily due to the H → L electronic transition with contributions of 68–90%. The corresponding spectra of the dye–Ti_5_O_10_ clusters have been presented in Fig. 13.

**Fig. 14:**
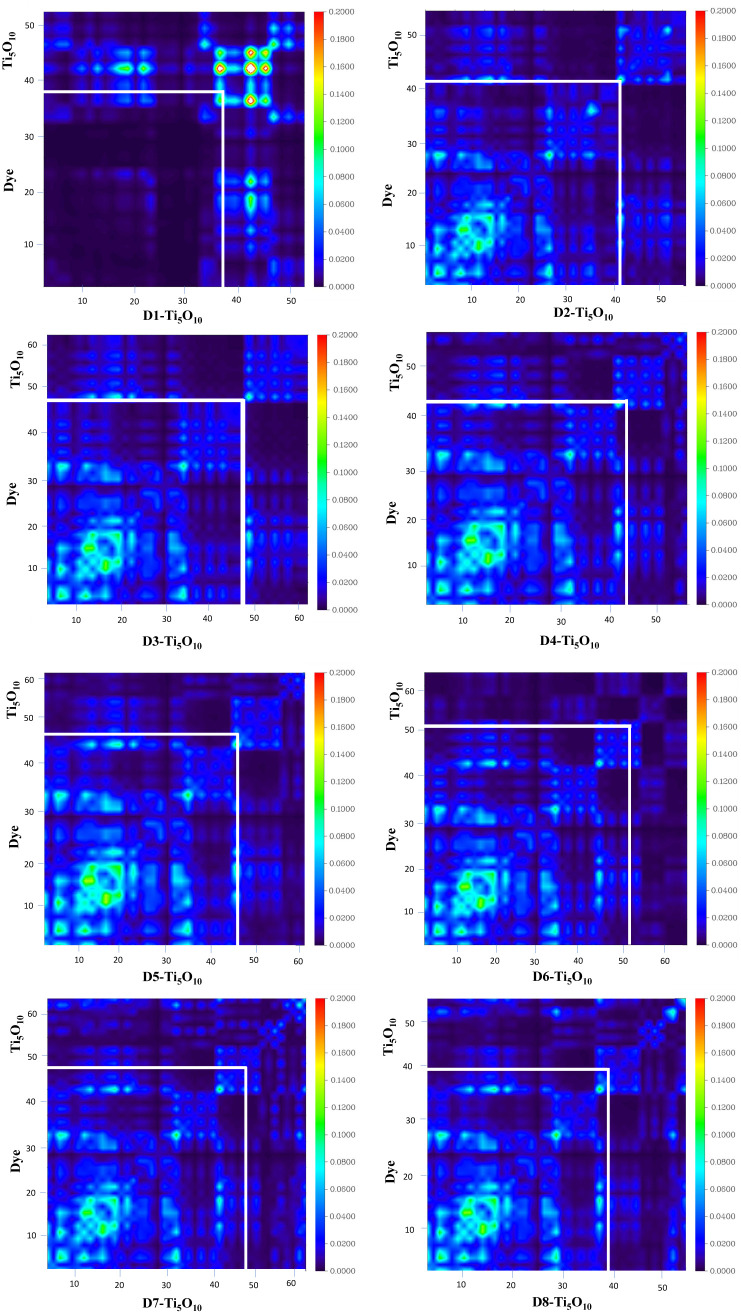
TDM plots of all the designed dye–Ti_5_O_10_ clusters.

For the TDM analysis of the dye clusters, all dye-cluster systems have been divided into two parts: the dye and the Ti_5_O_10_ cluster. The Multiwfn 3.8 program has been used to generate TDM plots for the dye clusters in the gas phase. Fig. 14 presents the TDM plots for the dye-clusters at an isosurface value of 0.002. The plots clearly show that the charge density is extensively distributed across both the dye and the Ti_5_O_10_ cluster, appearing predominantly along the diagonal. Analysis of all designed dyes confirms efficient electron transfer from the dye to the Ti_5_O_10_ cluster. The visual representation indicates a green region over the dye and a blue region over the Ti_5_O_10_ cluster, highlighting significant electron transfer from the dye to the TiO_2_ semiconductor surface.

The exciton binding energy (EBE) plays a crucial role in assessing the effectiveness of solar cells. It is directly related to the separation of charges within solar cells.^66^ The working principle of DSSCs involves the absorption of sunlight by dye molecules, leading to the generation of excitons (*i.e.*, electron–hole pairs). These excitons subsequently split upon reaching the surface of the semiconductor. In this context, EBE is defined as the amount of energy needed to separate the electron and hole from an exciton.^67,68^ This ease of charge separation is crucial in applications of dye-sensitized solar cells, where efficient conversion of light energy into electrical energy relies on effective charge transfer processes. It has been reported that the optimal value of *E*_b_ for organic semiconducting materials falls within the range of 0.2 to 1 eV.^69^ The expression for *E*_b_ can be written as *E*_b_ = *Δ*_H–L_ − *E*_1_, where *E*_1_ denotes the optical band gap.^69,70^ The EBE values have been reported in Table 11.

Table 11 indicates that all dye–Ti_5_O_10_ clusters have low *E*_b_ values, which in turn indicates easy charge separation. Among the eight dye–Ti_5_O_10_ clusters, the dye D1–Ti_5_O_10_ cluster has a lower *E*_b_ value. This suggests the electron transport from the dye to the Ti_5_O_10_ semiconducting surface will be easier in the D1 dye.

### 5 Conclusion

We have performed detailed theoretical investigations to gather information on the optoelectronic properties of eight groups of dyes that are designed based on the D–D–π–A architecture. To explore their structural and electronic properties, we have calculated the dihedral angles, *Δ*_H–L_ values and *µ* values of the designed dyes. From the observed values, it can be revealed that among all of the dyes, D1 and D7 possess lower *Δ*_H–L_ values. It is noteworthy that the GSOP values of all the designed dyes lie below the redox potential of the I^−^/I_3_^−^ electrolyte couple. Moreover, the ESOP values of the dyes lie above the conduction band of the TiO_2_ semiconducting surface. The acquired *λ*_tot_ values demonstrate the enhanced efficiency of electron–hole separation in the designed dyes. From the absorption properties, it is evident that all the designed dyes absorb sunlight in the visible regions. Thus, we can confidently assert that our designed dyes hold promise as suitable options for constructing DSSCs.

In order to assess the viability of our designed dyes, we have used the Ti_5_O_10_ surface. For dye–Ti_5_O_10_ clusters, it can be inferred that the *µ*_g_ values are higher and *Δ*_H–L_ values are lower than those of their isolated counterparts. This confirms the enhancement of the charge transport properties of the designed dyes upon binding the dye to the Ti_5_O_10_ semiconducting surface. Furthermore, the absorption spectra of the dye–Ti_5_O_10_ clusters demonstrate a rise in the *λ*_max_ values. This suggests the presence of a red shift in comparison to the isolated dyes. Consequently, the dyes exhibit enhanced performance upon binding to the TiO_2_ surface. In short, we can conclude that our designed dyes serve as viable options for the fabrication of DSSCs.

## Conflicts of interest

The authors have no conflicts to disclose.

## Supplementary Material

RA-015-D5RA07959D-s001

## Data Availability

The data supporting this article have been included as part of the supplementary information (SI). Supplementary information: optimized coordinates of the studied dyes, HOMO, LUMO, *Δ*_H–L_, and absorption energies of the test compound, the optimized structure of the test compound, and the justification for selecting Ti_5_O_10_ clusters have been provided. See DOI: https://doi.org/10.1039/d5ra07959d.
