# Supplementary material for: Tuning the auxiliary donor in D–D–π–A photosensitizers to enhance DSSC photovoltaic performance: a DFT/TDDFT study
Source: RSC Adv. 2025 Dec 17;15(59):50751–64. doi: 10.1039/d5ra07959d (PMC12709591; doi:10.1039/d5ra07959d)
Supplement: RA-015-D5RA07959D-s001 [file RA-015-D5RA07959D-s001.pdf]

Supplementary Information (SI) for RSC Advances.
This journal is © The Royal Society of Chemistry 2025

Supporting Information

Tuning the Auxiliary Donor in D-D-π-A Photosensitizers to Enhance DSSC Photovoltaic Performance: A DFT/TDDFT Study

Rinki Deka, Bikash Kalita and Dhruba Jyoti Kalita*
Department of Chemistry, Gauhati University, Guwahati-781014, India
E-mail: dhrubajyoti.kalita@gauhati.ac.in

Table S1 : Coordinates of the designed dyes in the angstrom unit.

1. Dye D1

Atoms	Coordinates (Angstroms)		
	X	Y	Z
C	2.345178	0.631825	-0.337120
C	1.179866	-0.126684	-0.223710
C	-0.090146	0.479455	-0.180311
C	2.217437	2.039268	-0.400536
C	0.978883	2.664527	-0.354414
C	-0.168932	1.881749	-0.247759
C	-2.580658	1.847207	-0.119253
C	-2.531632	0.374832	-0.004824
C	-1.314792	-0.249864	-0.056205
C	-3.784448	-0.357773	0.148895
C	-3.906692	-1.717956	0.417592
C	-5.234988	-2.153612	0.484620
S	-5.355816	0.395653	-0.006020
O	-1.375502	2.517011	-0.215922
O	-3.591568	2.511773	-0.137148
C	-7.575239	-1.364749	0.303500
C	-8.626214	-0.508561	0.120740
C	-8.463774	0.881835	-0.158126
C	-10.034586	-0.971853	0.197581
N	-8.309269	2.012649	-0.387083
O	-11.002545	-0.257739	0.043076
O	-10.141106	-2.301969	0.463076
H	-11.093710	-2.487280	0.489419

H	1.243422	-1.207621	-0.141060
H	3.109797	2.646082	-0.513539
H	0.879696	3.742471	-0.413226
H	-1.252564	-1.332798	-0.009372
H	-3.059713	-2.374226	0.574190
H	-5.528103	-3.177705	0.688334
H	-7.847915	-2.397087	0.504948
C	-6.164324	-1.137919	0.272469
C	3.677096	-0.013403	-0.395612
C	4.788721	0.575119	0.238138
C	6.033898	-0.025109	0.188166
C	6.227355	-1.237114	-0.501839
C	5.129949	-1.819054	-1.138654
C	3.872632	-1.216958	-1.084429
C	8.358407	0.138281	0.627093
C	8.596467	-1.065546	-0.058465
C	9.409801	0.904523	1.104883
C	10.728617	0.477244	0.912394
C	10.977042	-0.712545	0.232960
C	9.914444	-1.479879	-0.253625
N	7.497294	-1.807735	-0.503160
H	7.670191	-2.577186	-1.131815
H	4.692053	1.494001	0.806052
H	3.044212	-1.676394	-1.613735
H	5.266193	-2.747352	-1.687024
H	9.179139	1.827404	1.626086
H	11.548789	1.076956	1.292576
H	11.995589	-1.052493	0.075722
H	10.104158	-2.409470	-0.784062
O	7.069398	0.588943	0.866870

2. Dye D2

C	0.561398	1.282381	-0.235453
C	-0.519640	0.403260	-0.123779
C	-1.845219	0.872227	-0.105087
C	0.286292	2.669863	-0.309894
C	-1.011934	3.157849	-0.280252
C	-2.072104	2.258211	-0.184920
C	-4.467942	1.968435	-0.087036

C	-4.263336	0.511058	0.048392
C	-2.987444	0.017768	0.013686
C	-5.432396	-0.348526	0.204222
C	-5.411240	-1.703829	0.519473
C	-6.685242	-2.280947	0.573638
S	-7.072186	0.221696	-0.010211
O	-3.338949	2.762072	-0.174136
O	-5.543342	2.520558	-0.130339
C	-9.092514	-1.761707	0.314632
C	-10.225949	-1.034226	0.076949
C	-10.209905	0.354611	-0.252109
C	-11.576710	-1.647568	0.142639
N	-10.173520	1.486487	-0.521406
O	-12.612779	-1.050654	-0.059286
O	-11.542810	-2.970601	0.457305
H	-12.469497	-3.259679	0.471352
H	-0.342187	-0.663162	-0.025723
H	1.109040	3.369991	-0.414145
H	-1.223631	4.219074	-0.343983
H	-2.812669	-1.052230	0.070579
H	-4.501323	-2.255583	0.720080
H	-6.869977	-3.323362	0.809043
H	-9.255204	-2.810003	0.549471
C	-7.714364	-1.381673	0.304682
C	1.931328	0.767230	-0.282275
C	2.363158	-0.449939	-0.775119
C	3.760577	-0.625827	-0.651416
S	3.287510	1.711607	0.354139
H	1.687963	-1.162250	-1.235432
C	4.396809	0.451760	-0.067705
C	5.826519	0.233234	0.030764
C	6.079144	-1.049279	-0.526297
C	4.779584	-1.681937	-1.002284
C	7.367791	-1.560293	-0.580451
C	8.405016	-0.770626	-0.069133
C	8.151935	0.512416	0.487988
C	6.864041	1.023710	0.541923
C	9.452236	1.143224	0.963911
C	10.469698	0.084008	0.618294
C	9.835556	-0.991183	0.031048

C	11.874455	-0.091267	0.736346
C	12.280655	-1.301778	0.233868
S	10.949355	-2.249510	-0.393478
H	4.584820	-2.635783	-0.495474
H	4.805213	-1.895574	-2.078542
H	6.662860	2.003661	0.965999
H	7.569135	-2.539954	-1.004933
H	9.649205	2.095031	0.453890
H	9.424145	1.361693	2.039235
H	12.559468	0.629964	1.167030
H	13.283932	-1.702784	0.190388

3. Dye D3

C	1.037859	0.813545	1.172618
C	2.137955	0.089970	0.710498
C	3.444564	0.610113	0.781747
C	1.274457	2.098140	1.718040
C	2.549914	2.641167	1.791009
C	3.629327	1.894718	1.322759
C	6.014345	1.814284	0.949980
C	5.859529	0.451434	0.401192
C	4.603976	-0.087550	0.317643
C	7.051339	-0.264530	-0.042243
C	7.106104	-1.603055	-0.420002
C	8.379106	-2.011440	-0.834020
S	8.620996	0.493858	-0.186599
O	4.873249	2.450583	1.399164
O	7.060271	2.417523	1.033170
C	10.697025	-1.201321	-1.154606
C	11.760886	-0.341407	-1.174488
C	11.666755	1.029268	-0.787581
C	13.110937	-0.778064	-1.610395
N	11.568265	2.144853	-0.470042
O	14.087217	-0.059460	-1.648891
O	13.153206	-2.089333	-1.971412
H	14.072497	-2.257737	-2.234049
H	1.992499	-0.887840	0.260807
H	0.436981	2.665930	2.109893

H	2.730226	3.623651	2.212446
H	4.459009	-1.071912	-0.117093
H	6.252350	-2.268186	-0.383602
H	8.617304	-3.017888	-1.160504
H	10.914257	-2.214875	-1.480438
C	9.332880	-0.997501	-0.780644
C	-0.332832	0.258836	1.095953
C	-1.429115	1.078263	0.784938
C	-2.731091	0.582413	0.694271
C	-2.942111	-0.782322	0.915318
C	-1.856723	-1.616561	1.249407
C	-0.574299	-1.112302	1.333530
C	-3.892193	-2.808721	1.586456
C	-2.368661	-3.025190	1.452579
N	-4.127282	-1.517310	0.918977
C	-5.411184	-1.079082	0.570858
C	-6.543102	-1.592649	1.227880
C	-5.611895	-0.166495	-0.480253
C	-7.822863	-1.190878	0.860603
C	-8.033532	-0.256767	-0.166398
C	-6.895768	0.236090	-0.828276
C	-9.383826	0.233484	-0.535645
C	-11.918064	1.246227	-1.285475
C	-10.836183	2.117711	-1.154271
C	-9.596098	1.592442	-0.781805
C	-10.503347	-0.628190	-0.678567
C	-11.748431	-0.125681	-1.047772
O	-13.184768	1.616834	-1.635523
O	-10.276556	-1.956404	-0.452669
C	-13.430668	2.991471	-1.891940
C	-11.344639	-2.871975	-0.638294
H	-13.231589	3.610739	-1.007466
H	-14.487902	3.065611	-2.151013
H	-12.826583	3.361816	-2.730576
H	-11.721932	-2.850103	-1.668754
H	-12.175442	-2.673272	0.051260
H	-10.931180	-3.859385	-0.427302
H	-10.939283	3.182023	-1.322065
H	-8.759444	2.273216	-0.656709
H	-7.022980	0.922454	-1.660342

H	-4.763964	0.190266	-1.053011
H	-6.425012	-2.297622	2.043552
H	-8.676353	-1.600281	1.387507
H	-12.613444	-0.764104	-1.168536
H	-4.183220	-2.751687	2.646051
H	-4.488526	-3.591909	1.111471
H	-2.140049	-3.646104	0.576637
H	-1.943828	-3.521252	2.329190
H	-3.553592	1.250250	0.472222
H	0.246897	-1.764966	1.617299
H	-1.263465	2.133330	0.587836

4. Dye D4

C	0.732011	0.288689	-1.517450
C	-0.334771	-0.077579	-0.696203
C	-1.669014	0.179638	-1.064616
C	0.434304	0.934783	-2.740299
C	-0.870927	1.209642	-3.125481
C	-1.916526	0.830908	-2.286040
C	-4.304610	0.806188	-1.924906
C	-4.081601	0.097817	-0.647641
C	-2.797171	-0.170180	-0.257636
C	-5.240292	-0.279979	0.155143
C	-5.218474	-1.081859	1.292482
C	-6.478413	-1.249679	1.877762
S	-6.865694	0.259333	-0.202392
O	-3.190577	1.116089	-2.681860
O	-5.384289	1.139215	-2.358030
C	-8.861591	-0.626119	1.628581
C	-9.984314	-0.044439	1.106699
C	-9.969787	0.777495	-0.060407
C	-11.319917	-0.226072	1.728757
N	-9.935437	1.446472	-1.012435
O	-12.346119	0.266175	1.309914
O	-11.285381	-1.013549	2.837810
H	-12.202258	-1.059627	3.153556
H	-0.142999	-0.550462	0.262484
H	1.247450	1.204151	-3.406270

H	-1.098851	1.700530	-4.064848
H	-2.604063	-0.658102	0.692910
H	-4.318677	-1.543021	1.680109
H	-6.660228	-1.843219	2.767063
H	-9.021533	-1.222280	2.522810
C	-7.498393	-0.583713	1.202039
C	2.133125	0.014727	-1.122009
C	2.486484	-1.168179	-0.451680
C	3.159788	0.934337	-1.395128
C	4.474836	0.687146	-1.022539
C	4.817078	-0.495430	-0.342846
C	3.795576	-1.419942	-0.060438
C	6.970511	0.360117	0.461659
C	6.512075	1.285992	1.402114
C	8.258043	0.520396	-0.075274
C	9.062350	1.576759	0.325687
C	8.591059	2.513203	1.258652
C	7.305403	2.365865	1.793436
C	6.688791	-2.046394	0.083004
C	6.398210	-2.961972	-0.941339
C	6.940137	-4.244846	-0.908929
C	7.793141	-4.630974	0.127559
C	8.094417	-3.716993	1.139635
N	6.148799	-0.733383	0.056435
H	1.724406	-1.913113	-0.242090
H	4.037365	-2.340669	0.458454
H	2.923978	1.869493	-1.894677
H	5.246775	1.415661	-1.243786
H	6.916854	3.069162	2.519738
H	5.519460	1.165412	1.823837
H	10.059375	1.709147	-0.080582
H	8.753030	-4.003837	1.954292
H	8.220035	-5.628892	0.143655
H	6.705210	-4.941246	-1.708776
H	5.748272	-2.659840	-1.755581
O	9.456118	3.520715	1.573783
C	9.034939	4.495641	2.515588
H	9.866059	5.195221	2.616809
H	8.819675	4.047710	3.494950
H	8.146194	5.039530	2.167611

H	8.622023	-0.198189	-0.802172
C	7.543560	-2.437070	1.125604
H	7.771792	-1.731204	1.916608

5. Dye D5

C	0.476977	0.295601	-1.607732
C	1.557133	-0.221041	-0.890789
C	2.862295	0.279064	-1.062196
C	0.732809	1.349240	-2.517798
C	2.006463	1.870947	-2.698243
C	3.065742	1.333208	-1.970150
C	5.430085	1.423809	-1.485043
C	5.257040	0.297008	-0.545462
C	4.001964	-0.214368	-0.352295
C	6.430560	-0.221709	0.149767
C	6.473400	-1.372799	0.931076
C	7.728556	-1.609177	1.503142
S	7.989929	0.570257	0.111639
O	4.308338	1.864156	-2.160751
O	6.475164	1.992991	-1.705708
C	10.027815	-0.701806	1.644861
C	11.087959	0.137140	1.435754
C	11.007252	1.315895	0.634902
C	12.419279	-0.120390	2.039568
N	10.919356	2.274063	-0.020270
O	13.391286	0.589429	1.890034
O	12.449325	-1.252571	2.793818
H	13.357044	-1.316196	3.132115
H	1.395655	-1.011645	-0.163900
H	-0.086633	1.745777	-3.108165
H	2.201283	2.675343	-3.398517
H	3.841914	-1.017256	0.360989
H	5.624356	-2.030081	1.071443
H	7.955858	-2.458162	2.138599
H	10.233413	-1.558443	2.280928
C	8.680419	-0.647532	1.171861
C	-0.891621	-0.239495	-1.427114
C	-2.010587	0.609139	-1.453799

C	-3.309719	0.135420	-1.267983
C	-3.495998	-1.232008	-1.038914
C	-2.389583	-2.102328	-1.035038
C	-1.108968	-1.619569	-1.224632
C	-4.361627	-3.338841	-0.488850
C	-2.885327	-3.525443	-0.907668
N	-4.681971	-1.942748	-0.868735
C	-5.920283	-1.388950	-0.468896
C	-7.109278	-1.984431	-0.915654
C	-6.006577	-0.286679	0.395013
C	-8.344515	-1.486869	-0.517109
C	-8.444976	-0.371329	0.332690
C	-7.247515	0.213404	0.777577
C	-9.762668	0.165134	0.743772
C	-12.279020	1.189443	1.526930
C	-11.213127	2.060585	1.273322
C	-9.976279	1.542841	0.885796
C	-10.849865	-0.690910	1.005947
C	-12.086134	-0.193085	1.389257
O	-13.530359	1.576889	1.908480
C	-13.783087	2.964452	2.071885
H	-13.142033	3.405225	2.846776
H	-14.826265	3.046605	2.380434
H	-13.640504	3.516358	1.133389
H	-11.334464	3.133483	1.360312
H	-9.168263	2.233477	0.663350
H	-7.288352	1.049518	1.469313
H	-5.099637	0.160959	0.786289
H	-7.057638	-2.825252	-1.600243
H	-9.249557	-1.946369	-0.902692
H	-12.919961	-0.854144	1.600725
H	-5.035800	-4.041777	-0.983596
H	-4.487243	-3.457935	0.596882
H	-2.325960	-4.120713	-0.180195
H	-2.814245	-4.039778	-1.874236
H	-4.151429	0.817047	-1.296836
H	-0.268825	-2.308438	-1.249440
H	-1.863969	1.676166	-1.592965
H	-10.713314	-1.765665	0.932532

6. Dye D6

C	-0.938827	-1.437464	0.552306
C	-1.984787	-0.566422	0.246005
C	-3.328092	-0.984285	0.297331
C	-1.265994	-2.765451	0.913665
C	-2.581179	-3.206820	0.968104
C	-3.605966	-2.313585	0.662199
C	-5.988835	-1.969386	0.465191
C	-5.730266	-0.578022	0.040429
C	-4.436658	-0.134086	-0.011625
C	-6.865995	0.274298	-0.296518
C	-6.793954	1.542761	-0.864662
C	-8.045919	2.141657	-1.046011
S	-8.526826	-0.188461	-0.000568
O	-4.890928	-2.765533	0.733902
O	-7.084167	-2.465664	0.598464
C	-10.472267	1.763475	-0.724669
C	-11.632272	1.132519	-0.367881
C	-11.667486	-0.168652	0.217898
C	-12.960052	1.767130	-0.564077
N	-11.673945	-1.229679	0.696663
O	-14.017735	1.254108	-0.265958
O	-12.878346	3.005394	-1.121591
H	-13.794371	3.317062	-1.200641
H	-1.768163	0.451772	-0.063177
H	-0.468709	-3.450976	1.181419
H	-2.833062	-4.221180	1.256088
H	-4.221259	0.894309	-0.285110
H	-5.863434	2.016007	-1.152405
H	-8.190856	3.125901	-1.477769
H	-10.596390	2.753143	-1.155714
C	-9.108822	1.345897	-0.624970
C	0.472443	-0.989057	0.504114
C	1.486255	-1.843123	0.029322
C	2.797368	-1.403525	-0.008618
C	3.163146	-0.104927	0.417609
C	0.835688	0.298993	0.932631
C	2.155754	0.744648	0.893543

C	4.617433	0.065631	0.253630
C	5.120705	-1.145978	-0.275524
C	4.018598	-2.150236	-0.483089
C	6.499814	-1.265435	-0.528753
C	7.269821	-0.148429	-0.227485
C	5.454115	1.217338	0.550782
C	6.849142	1.072298	0.288021
C	5.061246	2.473565	1.068475
C	6.009368	3.465976	1.290968
C	7.379963	3.288398	1.022320
C	7.849421	2.080827	0.508482
C	9.277728	1.783690	0.182544
C	9.615463	0.496109	-0.345993
C	8.594707	-0.423881	-0.531519
C	10.919374	0.065060	-0.706896
C	11.960235	1.002988	-0.518164
C	10.353959	2.656974	0.341721
C	11.664544	2.260954	-0.005682
C	8.694540	-1.716527	-1.030412
C	9.992761	-2.159288	-1.395214
C	11.053538	-1.273848	-1.227002
N	7.387673	-2.237188	-1.026694
H	1.234549	-2.836616	-0.332091
H	3.931775	-2.451303	-1.536117
H	4.186731	-3.069785	0.094058
H	0.071186	0.957556	1.334269
H	2.375607	1.742219	1.249267
H	4.026236	2.687607	1.296687
H	5.679599	4.421318	1.689166
H	8.062196	4.108955	1.221284
H	10.202408	3.657246	0.735638
H	12.471607	2.974143	0.135364
H	12.984527	0.743582	-0.771996
H	12.049522	-1.607973	-1.504632
H	10.175798	-3.152102	-1.794713
H	7.128484	-3.160438	-1.333248

7. Dye D7

C	-0.248504	-0.964051	-1.323694
C	0.793659	-0.436295	-0.560730
C	2.141130	-0.700539	-0.872516
C	0.088969	-1.782592	-2.427808
C	1.408064	-2.065061	-2.755661
C	2.428055	-1.521256	-1.977774
C	4.809735	-1.319883	-1.630346
C	4.543360	-0.482213	-0.442938
C	3.245356	-0.192679	-0.118198
C	5.676149	0.011494	0.333491
C	5.601332	0.697420	1.542439
C	6.851772	1.071673	2.047088
S	7.338636	-0.184376	-0.175471
O	3.717254	-1.804583	-2.323569
O	5.908634	-1.612745	-2.044439
C	9.279160	0.956746	1.571539
C	10.441999	0.658211	0.915526
C	10.482533	-0.046161	-0.325260
C	11.767163	1.054504	1.454241
N	10.493913	-0.620294	-1.337833
O	12.827547	0.817387	0.915394
O	11.680606	1.724217	2.635565
H	12.595778	1.928649	2.886809
H	0.572098	0.173246	0.310309
H	-0.705080	-2.181046	-3.050838
H	1.666065	-2.684464	-3.607208
H	3.022551	0.445420	0.731521
H	4.669591	0.911737	2.050877
H	6.994071	1.609831	2.977906
H	9.400286	1.500206	2.504590
C	7.917088	0.679370	1.239813
C	-1.663531	-0.679043	-0.994331
C	-2.066687	0.583718	-0.529238
C	-2.658020	-1.662011	-1.133026
C	-3.987887	-1.401046	-0.830516
C	-4.382050	-0.134590	-0.358467
C	-3.391158	0.854107	-0.209520
C	-6.585470	-0.900428	0.425909
C	-6.182995	-1.752035	1.458712
C	-7.864973	-1.068985	-0.128378

C	-8.713906	-2.059200	0.343103
C	-8.298370	-2.921785	1.368850
C	-7.022024	-2.766878	1.922736
C	-6.260043	1.448666	-0.178901
C	-6.033838	2.203422	-1.342655
C	-6.564106	3.478360	-1.471257
C	-7.357434	4.024384	-0.450693
C	-7.601794	3.274318	0.705324
N	-5.727261	0.133946	-0.047860
H	-1.331978	1.377590	-0.429957
H	-3.669491	1.839052	0.147546
H	-2.384825	-2.657247	-1.471607
H	-4.731354	-2.180758	-0.949974
H	-6.675256	-3.412616	2.720239
H	-5.198066	-1.625929	1.896331
H	-9.704517	-2.196095	-0.077267
H	-8.209850	3.668482	1.510352
H	-6.393324	4.069315	-2.364790
H	-5.432537	1.781567	-2.141164
O	-9.205721	-3.868596	1.749890
C	-8.841307	-4.766022	2.787267
H	-9.696650	-5.428478	2.927732
H	-8.636452	-4.237893	3.728075
H	-7.962122	-5.365647	2.515906
H	-8.187361	-0.409088	-0.926990
C	-7.043867	2.001298	0.837586
H	-7.228822	1.423343	1.737036
O	-7.839958	5.280357	-0.684974
C	-8.651755	5.882406	0.311441
H	-9.565563	5.302017	0.496465
H	-8.924405	6.865635	-0.074762
H	-8.109243	6.005266	1.258372

8. Dye D8

C	-2.580220	-0.610846	-0.545253
C	-1.402306	0.049643	-0.190456
C	-0.144972	-0.530657	-0.443248

C	-2.482770	-1.886005	-1.155373
C	-1.252882	-2.482212	-1.387196
C	-0.087049	-1.804229	-1.034028
C	2.325669	-1.844818	-0.956236
C	2.303700	-0.491585	-0.363932
C	1.095364	0.101578	-0.113512
C	3.573299	0.159674	-0.058697
C	3.736775	1.483193	0.340502
C	5.069411	1.825643	0.595342
S	5.112489	-0.668241	-0.135790
O	1.110863	-2.414869	-1.276375
O	3.325343	-2.491297	-1.178869
C	7.369840	0.910835	0.594961
C	8.388063	0.006599	0.461931
C	8.184103	-1.349395	0.065779
C	9.800567	0.375616	0.728798
N	7.996257	-2.452188	-0.256195
O	10.740832	-0.384199	0.629920
O	9.947575	1.675600	1.103777
H	10.899508	1.798417	1.249236
H	-1.448655	1.026383	0.278179
H	-3.391351	-2.401490	-1.443951
H	-1.176656	-3.458576	-1.852261
H	1.052464	1.080472	0.354209
H	2.916740	2.184573	0.431097
H	5.392879	2.811386	0.911545
H	7.672566	1.905852	0.909609
C	5.961643	0.774531	0.395545
C	-4.963706	-0.839347	0.016985
C	-4.857467	-1.860459	0.965694
C	-6.208343	-0.610685	-0.593165
C	-7.310696	-1.381748	-0.257639
C	-7.194303	-2.417870	0.682133
C	-5.956772	-2.658343	1.290037
C	-3.993611	1.388457	-0.312244
C	-3.480623	2.169711	-1.361888
C	-3.631776	3.547998	-1.354844
C	-4.323840	4.181616	-0.310824
C	-4.852724	3.410377	0.730360
N	-3.842157	-0.028583	-0.323205

H	-5.836640	-3.446282	2.023304
H	-3.903265	-2.040362	1.450262
H	-8.276285	-1.211946	-0.721854
H	-5.392355	3.870053	1.549256
H	-3.238616	4.159600	-2.159925
H	-2.957965	1.683903	-2.179359
O	-8.336333	-3.123486	0.928334
C	-8.279794	-4.183119	1.871690
H	-9.284669	-4.605810	1.911055
H	-8.000988	-3.822756	2.870829
H	-7.572160	-4.964019	1.562930
H	-6.300523	0.183321	-1.326819
C	-4.673733	2.025373	0.728843
H	-5.078597	1.429680	1.540273
O	-4.425576	5.539356	-0.408459
C	-5.122786	6.234259	0.614633
H	-6.170561	5.912505	0.681897
H	-5.088800	7.289453	0.339808
H	-4.644606	6.101350	1.594300

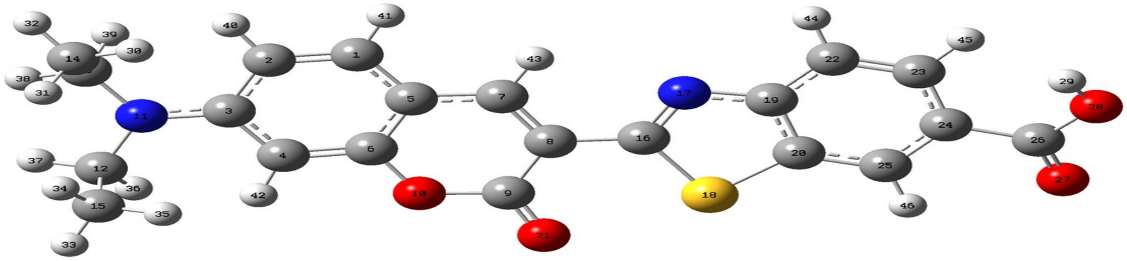

Fig. S1: Optimized structure of the test compound.

Table S2: Energies of HOMO, LUMO, Δ_{H-L}, and λ_{max} values of the test compound with different functional.

**Functional**	**HOMO (eV)**	**LUMO (eV)**	**Δ_{H-L} (eV)**	**λ_{max} (nm)**
Experimental	−5.17	−2.67	2.50	343
B3LYP	-5.24	-2.91	2.33	607
B3LYP-D3	-5.26	-2.90	2.36	610
CAM-B3LYP	-6.47	-1.73	4.74	375
B3PW91	-5.33	-3.00	2.33	607
WB97XD	-7.04	-1.18	5.86	300
HSEH1PBE	-5.09	-3.16	1.93	646

To provide the necessary validation for chosing Ti_5O_{10} cluster, we have carried out literature survey and selected one publication where Ti_9O_{18} cluster has been used throughout their work (Phys. Chem. Chem. Phys., 2012, 14, 225–233). In this regard, we have chosen one of their reported dye (viz., NKX-2753) and anchored the same with our used Ti_5O_{10} cluster (in our manuscript) and carried out the optimization employing the same level of theory. The optimized structure of our designed NKX-2753-Ti_5O_{10} dye-cluster is presented in Fig. S2.

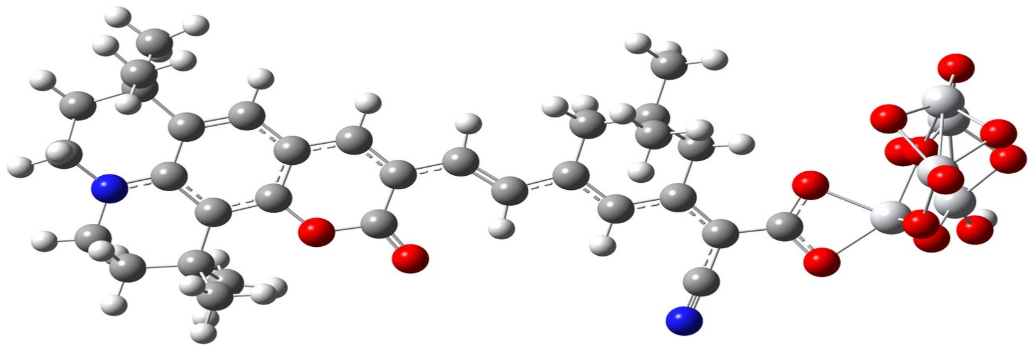

Fig. S2: Optimized structure of the NKX-2753-Ti_5O_{10} dye-cluster.

Here, our aim is to compare the calculated energy band gap (Δ_{H-L}) values of both the reported dye-cluster (NKX-2753-Ti_9O_{18}) and our designed NKX-2753-Ti_5O_{10} cluster. The respective Δ_{H-L} values of both the dye-clusters are reported in the Table S3:

Table S3: Validation of basis set.

Dye-cluster	Δ_{H-L} (eV)
NKX-2753-Ti_9O_{18}	1.319
NKX-2753- Ti_5O_{10}	1.317

From Table S3, it is evident that both the dye-clusters exhibit nearly the same Δ_{H-L} value. Hence, we can conclude that our used Ti_5O_{10} cluster is sufficient for the DFT and TD-DFT simulations.
